# Metabolic impact of genetic and chemical ADP/ATP carrier inhibition in renal proximal tubule epithelial cells

**DOI:** 10.1007/s00204-023-03510-7

**Published:** 2023-05-08

**Authors:** Charlotte A. Hoogstraten, Maaike M. E. Jacobs, Guido de Boer, Melissa A. E. van de Wal, Werner J. H. Koopman, Jan A. M. Smeitink, Frans G. M. Russel, Tom J. J. Schirris

**Affiliations:** 1 Department of Pharmacology and Toxicology, Radboud Institute for Molecular Life Sciences, Radboud University Medical Center, Nijmegen, 6500 HB The Netherlands; 2 Radboud Center for Mitochondrial Medicine, Radboud University Medical Center, Nijmegen, 6500 HB The Netherlands; 3 Department of Pediatrics, Amalia Children’s Hospital, Radboud University Medical Center, Nijmegen, 6500 HB The Netherlands; 4 Khondrion BV, Nijmegen, 6525 EX The Netherlands

**Keywords:** Nephrotoxicity, Drug-induced mitochondrial dysfunction, ADP/ATP carrier, Off-target, CRISPR/Cas9, Oxidative metabolism

## Abstract

Mitochondrial dysfunction is pivotal in drug-induced acute kidney injury (AKI), but the underlying mechanisms remain largely unknown. Transport proteins embedded in the mitochondrial inner membrane form a significant class of potential drug off-targets. So far, most transporter-drug interactions have been reported for the mitochondrial ADP/ATP carrier (AAC). Since it remains unknown to what extent AAC contributes to drug-induced mitochondrial dysfunction in AKI, we here aimed to better understand the functional role of AAC in the energy metabolism of human renal proximal tubular cells. To this end, CRISPR/Cas9 technology was applied to generate *AAC3*^−/−^ human conditionally immortalized renal proximal tubule epithelial cells. This *AAC3*^*−/−*^ cell model was characterized with respect to mitochondrial function and morphology. To explore whether this model could provide first insights into (mitochondrial) adverse drug effects with suspicion towards AAC-mediated mechanisms, wild-type and knockout cells were exposed to established AAC inhibitors, after which cellular metabolic activity and mitochondrial respiratory capacity were measured. Two *AAC3*^−/−^ clones showed a significant reduction in ADP import and ATP export rates and mitochondrial mass, without influencing overall morphology. *AAC3*^−/−^ clones exhibited reduced ATP production, oxygen consumption rates and metabolic spare capacity was particularly affected, mainly in conditions with galactose as carbon source. Chemical AAC inhibition was stronger compared to genetic inhibition in *AAC3*^*−/−*^, suggesting functional compensation by remaining AAC isoforms in our knockout model. In conclusion, our results indicate that ciPTEC-OAT1 cells have a predominantly oxidative phenotype that was not additionally activated by switching energy source. Genetic inhibition of AAC3 particularly impacted mitochondrial spare capacity, without affecting mitochondrial morphology, suggesting an important role for AAC in maintaining the metabolic spare respiration.

**Supplementary Information:**

The online version contains supplementary material available at 10.1007/s00204-023-03510-7.

## Introduction

Acute kidney injury (AKI) is a known major disease burden, affecting 20% of hospitalized adults worldwide (Ishimoto and Inagi 2016; Siew and Davenport 2015). Mortality rates are over 50 percent in severe cases and less severe manifestations are associated with chronic kidney disease (Hoste et al. 2015). A steadily increasing number of commonly used drugs have been associated with AKI (Palevsky et al. 2013) and various prospective cohort studies indicated that drug-induced mechanisms explain 14–26% of all AKI cases, emphasizing the importance of renal toxicity assessment (Mehta et al. 2004; Moffett and Goldstein 2011; Perazella 2018). Besides a major burden for the healthcare system, drug-induced AKI also has implications for the pharmaceutical industry as drug attrition in development, clinical and post-marketing phases is often due to renal toxicity (Gai et al. 2020; Hoste et al. 2015; Mehta et al. 2004; Uchino et al. 2005; van der Meer et al. 2010).

Mitochondrial dysfunction has shown to play a pivotal role in AKI (Bhatia et al. 2020). This is compatible with the kidneys being among the most energy-demanding organs in the human body, consuming approximately 10 percent of the total oxygen supply. Accordingly, tubular cells are mitochondria rich, to meet ATP demands (*e.g.* to sustain activity of ATP-consuming pumps and transporters) required for physiological function. In particular, the proximal tubule contains a large amount of mitochondria and is characterized by the relatively high protein expression levels of solute transporters (Gai et al. 2020; Huls et al. 2008; Koepsell 2013; Launay-Vacher et al. 2006; Motohashi and Inui 2013; Prasad et al. 2016; Smeets et al. 2004; Soltoff 1986). The latter facilitate high transport rates of solutes, drugs and their (toxic) metabolites, contributing to high intracellular drug exposure. This could augment the probability of drug-induced mitochondrial dysfunction, eventually resulting in renal insufficiency caused by proximal tubular cell death through ATP depletion and increased levels of oxidative stress (Gai et al. 2020; Heidari 2019).

The inner mitochondrial membrane (IMM) is virtually impermeable to most molecules and ions, which is essential for maintaining the proton gradient and the ensuing inward-directed proton-motive force to drive ATP generation by the oxidative phosphorylation system (OXPHOS). However, to link cytosolic and mitochondrial metabolic pathways, molecules, inorganic ions and cofactors need to be exchanged between both compartments. This is facilitated by a plethora of transport proteins embedded in the IMM (Jaiquel Baron et al. 2021; Kunji et al. 2020; Palmieri et al. 2020; Ruprecht and Kunji 2020), which account for more than five percent of the mitochondrial proteome. In principle, these transporters represent a significant class of potential drug off targets, which have largely been unexplored. Most of these transporters belong to the mitochondrial carrier family (SLC25) and enable import or efflux of solutes important for many cellular processes (Kunji 2011; Kunji et al. 2020; Palmieri and Monne 2016). In addition, other transporters are located on the IMM include the pyruvate carrier (Herzig et al. 2012; Kunji 2011), five ATP-binding cassette (ABC) transporters (Lill and Kispal 2001), a Mg^2+^-transporter, a Na^+^/Ca^2+^-exchanger, a Ca^2+^-uniporter, an aquaporin and five sideroflexins (Kunji 2011; Palmieri and Pierri 2010). The SLC25 family consists of a vast number of isoforms which transport the same substrates but vary in expression from ubiquitous to organ- or disease-specific patterns (Gutierrez-Aguilar and Baines 2013; Haitina et al. 2006; Palmieri 2004). A canonical member of the SLC25 family is the mitochondrial ADP/ATP carrier (AAC) that mediates electrogenic exchange of ADP^3−^ from the cytosol into the mitochondrial matrix against ATP^4−^ to supply energy to the cytosol (Klingenberg 2008; Kunji et al. 2016a; Ruprecht et al. 2019; Ruprecht and Kunji 2019). In humans four AAC isoforms are identified, which are encoded by the *SLC25A4*, *SLC25A5*, *SLC25A6* and *SLC25A31* genes in a tissue-specific manner. They cycle between a cytoplasmic- and matrix-open state, to enable bidirectional access to the substrate binding site. Due to their central role in cellular energy metabolism and more specifically in ADP/ATP exchange, they form a potential off-target in drug-induced mitochondrial dysfunction underlying proximal tubular toxicity and AKI (Jaiquel Baron et al. 2021).

In this respect, inhibition of AAC has previously been linked to mitochondrial dysfunction, *e.g.,* for the most potent prototypical AAC inhibitors bongkrekic acid (BKA) and carboxyatractyloside (CATR) and its precursor atractyligenin (Luciani et al. 1971; Vignais et al. 1966). Moreover, commonly used drugs, including furosemide, aspirin, diclofenac, sertraline and anthralin have been described to reduce mitochondrial ADP-uptake through AAC inhibition (Jaiquel Baron et al. 2021; Li et al. 2012; Manuel and Weiner 1977; Moreno-Sanchez et al. 1999; Salet et al. 1991). Also, ibipinabant and leelamine, two potential anti-obesity drugs, have been associated with AAC inhibition in preclinical drug development studies (Schirris et al. 2015; Zhang et al. 2016).

Here, we aimed to better understand the function of AAC in cellular energy metabolism in human conditionally immortalized proximal tubule epithelial cells (ciPTEC). To investigate this, we applied CRISPR/Cas9 to generate *AAC3*^*−/−*^ cells, the most abundant AAC isoform expressed in ciPTEC (Palmieri 2004; Stepien et al. 1992). This *AAC3*^*−/−*^ cell model was subsequently characterized based on function and morphology. Previously, overexpression models in *Lactococcus lactis, Saccharomyces cerevisiae* or proteoliposomes have been developed to study drug-induced AAC inhibition (Jaiquel Baron et al. 2021; Todisco et al. 2016; Zhang et al. 2016). However, these models overexpress the protein of interest without considering the complete and complex human cellular physiology, and the potential interactions between AAC and other mitochondrial or cellular pathways. To explore whether our cell model could be useful in providing insights into (mitochondrial) adverse effects of drugs with a suspicion towards AAC-mediated mechanisms, we exposed wild-type and *AAC3*^*−/−*^ cells to established AAC inhibitors and measured cellular metabolic activity and respiratory capacity.

## Materials and methods

### Compounds

Bongkrekic acid (BKA; #B6179), carboxyatractyloside potassium salt (CATR; #C4992), antimycin A (# A8674), oligomycin (#O4876), rotenone (#R8875) and carbonyl cyanide 4-(trifluoromethoxy) phenylhydrazone (FCCP; #C2920) were all purchased from Sigma-Aldrich (Zwijndrecht, The Netherlands). Suramin sodium (#SC-200833) was from Santa Cruz Biotechnologies (Santa Cruz, CA, USA) and CD437 (#HY-100532) was obtained from MedChem Express (Monmouth Junction, NJ, USA). [^14^C]-ADP (1 µCi, #NEC559050UC) was purchased from Perkin Elmer (Waltham, MA, USA).

### Cell culture

Human conditionally immortalized proximal tubule epithelial cells expressing organic anion transporter 1 (ciPTEC-OAT1, RRID:CVCL_LI01) were obtained and cultured as previously described (Nieskens et al. 2016; Wilmer et al. 2010). Proliferating cells were cultured at 33 °C and 5% (*v/v*) CO_2_ in 1:1 (*v/v*) Dulbecco’s modified Eagle’s medium and nutrient mixture F-12 with 17.5 mM glucose, 0.5 mM sodium pyruvate, 2.5 mM glutamine and without phenol red (DMEM Ham’s F-12, Life Technologies, Paisley, UK), supplemented with 5 µg/mL insulin, 5 µg/mL transferrin, 5 ng/mL selenium, 36 ng/mL hydrocortisone, 10 ng/mL human epidermal growth factor (EGF), 40 pg/mL trio-iodothyrine (all purchased from Sigma-Aldrich) and 10% (*v/v*) fetal bovine serum (FBS, Greiner Bio-One, Alpen a/d Rijn, The Netherlands), referred to as PTEC complete medium, which was refreshed every 2–3 days. Experimental cells proliferated for one day at 33 °C and 5% (*v/v*) CO_2_, followed by maturation at 37 °C and 5% (*v/v*) CO_2_ in PTEC complete medium for seven days to differentiate into an epithelial monolayer. Cells in experiment varied in passage numbers from 56 to 78.

### Single guide RNA design

Single guide RNAs (sgRNAs) targeting exon 1 and 2 of the *Homo sapiens* gene *AAC3* (*SLC25A6*) were designed for clustered regularly interspaced short palindromic repeat (CRISPR/Cas9) using CHOPCHOP and crispor.tefor.net (hg38/GRCh38 was used as reference genome). sgRNAs were selected based on predicted efficiency score and absence of off-targets with > 3 mismatches (Table 1). The oligonucleotide pair for each sgRNA was phosphorylated and annealed using T4 Polynucleotide Kinase (EK0031, Thermofisher Scientific) in a thermocycler at 37 °C for 30 min, followed by 5 min at 95 °C and 5 °C/min decrease to 25 °C, according to the manufacturer’s instructions. Plasmid pX333-GFP (kindly provided by Eric Verschuren, Dept. of Physiology, Radboudumc, Addgene, #64,073) was linearized at 37 °C for 1 h using *BbsI* (R0539, New England Biolabs, Ipswich, MA, USA), or *BsaI* (R0535, New England Biolabs) restriction enzymes. Ligation of annealed oligonucleotides into the linearized pX333-GFP plasmid was performed using T4 ligase (New England Biolabs) overnight at 16 °C, following instructions by the manufacturer. Heat shock at 42 °C transformed the ligation mix into TOP10F competent cells. The next day, colonies with pX333 containing sgRNAs were inoculated and plasmids were isolated using GenEluteTM Plasmid Miniprep Kit (Sigma-Aldrich) according to manufacturer’s instructions. As the plasmid enables dual expression of sgRNAs, sequencing resulted in selection of plasmids containing both sgRNAs (forward primer exon 1/2 sgRNA, see Table 1). Insertion of T2A-eGFP after NLS-Cas9-NLS, allowed GFP-positive FACS sorting after transfection. Prior to transfection ciPTEC-OAT1 exons 1–4 were sequenced to confirm wild-type sequences (Table 1).

**Table 1:** Primers for CRISPR/Cas9 gene editing

Gene	Forward (5′–3′)	Reverse (5′–3′)
Wild-type sequence validation		
*AAC3*—exon 1	GACGCCCTCCATTCACTCTC	GATCGCGGCCTTCCACTT
*AAC3*—exon 2	CCTGGGTCTGGTCTGAACAC	GAGCCCGTCTCTGGGACTT
*AAC3*—exon 3	CTCTCGTGTTGTAAACGTCAGC	GGGATTTAGAAACTGCCGCC
*AAC4*—exon 4	CTCTCGTGTTGTAAACGTCAGC	ACGTGGTTCTCTTGGTTCCC
CRISPR/Cas9		
*AAC3*—exon 1 sgRNA	caccgCGGCCGTGGCTCCGATCGAG	aaacCTCGATCGGAGCCACGGCCGc
*AAC3*—exon 2 sgRNA	caccgCGGCGTGTACGATACGGCCA	aaacTGGCCGTATCGTACACGCCGc
*AAC3*^*−/−*^ clone validation		
Amplification exon 1	CGGCCTGACCTTCACAAGG	AATCCGTGCCGCATTTCC
Sanger sequencing	GACGCCCTCCATTCACTCTC	GATCGCGGCCTTCCACTT
T7 endonuclease	GACGCCCTCCATTCACTCTC	GAGCCCGTCTCTGGGACTT

Lower case letters indicate 5′ overhangs, required for cloning of oligonucleotides into the PX333 plasmid

### CRISPR/Cas9-mediated genome editing

750,000 ciPTEC-OAT1 cells/well were seeded into a 6-wells plate and proliferated for 24 h at 33 °C and 5% *v/v* CO_2_. Cells were transfected with 2.5 μg of the isolated plasmid containing the sgRNAs using Mirus TransIT-X2 or LT1 reagent (MirusBio, Madison, WI, USA, 1:2 plasmid:reagent ratio (*v/v*)) in serum-free pTEC complete medium according to the manufacturer’s instructions. GFP-positive cells were single cell sorted into 96-wells plates by fluorescence-activated cell sorting (FACS, Aria Flow cytometer, BD Biosciences, San Jose, CA, USA) 40 h post-transfection and single cell clones were grown at 33 °C and 5% *v/v* CO_2_ in pTEC CM. Upon ~ 90% confluency, genomic DNA was isolated using prepGEM™ Universal DNA extraction kit (MicroGEM, Aotearoa, New Zealand) according to manufacturer’s protocol. Genome editing efficiency was assessed by T7 endonuclease mismatch detection assay, as described by the manufacturer. As inefficient targeting of exon 2 by sgRNA occurred, this region was not considered further. The targeted region in *AAC3* exon 1 was amplified using primer pairs (Table 1) and phire hot start II DNA polymerase (F-122S, Thermofisher Scientific) in a thermocycler (98 °C for 1 min and 40 cycles of 98 °C for 5 s, 68 °C for 5 s, and 72 °C for 15 s followed by a final elongation step of 72 °C for 1 min). PCR products were ExoSAP-IT (78,200, Applied Biosystems, Bleijswijk, The Netherlands), Sanger sequenced (primers see Table 1) and analyzed using SnapGene® Viewer (version 4.3.10, GSL Biotech LLC, San Diego, CA, USA) or Chromas Lite (version 2.1.1, Technelysium Pty Ltd, Australia). Both *AAC3*^*−/−*^ clones used in this study were generated independently.

### Real-time quantitative PCR

mRNA was isolated from matured ciPTEC-OAT1 cells using the RNeasy mini kit (Qiagen, Hilden, Germany) according to manufacturer’s instructions. Complementary DNA (cDNA) was synthesized using Molony-Murine Leukemia Virus (M-MLV) reverse transcriptase (Invitrogen, 28025013), as described in the manufacturer’s protocol. Gene expression levels of solute carrier (SLC) family members *SLC25A4* (ADP/ATP carrier 1: AAC1), *SLC25A5* (AAC2), *SLC25A6* (AAC3) and *SLC25A31* (AAC4) were measured by real-time quantitative PCR (RT-qPCR) using a CFX96-Touch Real-Time PCR system (BioRad Laboratories, Veenendaal, The Netherlands) and analyzed using BioRad CFX Manager (version 3.1). *GAPDH* levels were used as reference. Relative gene expression was determined by *GAPDH* subtraction, followed by calculation of 2^−ΔCt^. TaqMan Universal PCR Master Mix (Life Technologies, 4304437) and gene specific primer–probe sets (*SLC25A4*: Hs00154037_m1, *SLC25A5*: Hs00854499_g1, *SLC25A6*: Hs00745067_s1, *SLC25A31*: Hs00935856_m1 and *GAPDH*: Hs99999905_m1) were purchased from Applied Biosystems (Thermofisher Scientific). Non-template samples served as control.

### Western blot

To identify mitochondrial protein abundance of AAC1, AAC2 and AAC3 in ciPTEC-OAT1 wild type and *AAC3*^*−/−*^, Western blotting was performed. AAC4 was excluded from further analysis due to tissue-specific expression in the testis and absence of mRNA expression levels in our cell model. Cells were seeded at 63,000 cells/cm^2^ in T175 cell culture flasks and matured as described before. Mature cells were pelleted and lysed in RIPA buffer (50 mM Tris–HCl, 50 mM NaCl, 1% Triton X-100, 5 mM Na_2_-EDTA, 10 mM Na_4_P_2_O_7_.10H_2_O, 50 mM NaF, protease inhibitor and phosphatase inhibitor, pH 7.4 and 100 µg/ml DNase) on ice for 30 min, followed by centrifugation for 10 min at 4 °C and 6000 g. Protein was determined using the Protein Assay Dye Reagent Concentrate (500–0006, BioRad) according to the manufacturer’s instructions. Samples were incubated for 5 min at 95 °C in Laemmli sample buffer, after which 30 μg protein was loaded on a 4–15% (*w/v*) Mini-PROTEAN® TGX stain-free pre-cast SDS-PAGE gel (456-8024S, BioRad) for electrophoresis. Samples ran for 30 min at 50 V, followed by 45 min at 150 V and were transferred to a polyvinylidene difluoride (PVDF) membrane (0.45 μm) using the Trans-Blot® Turbo™ Transfer system (1704275, RTA transfer kit, BioRad) according to the manufacturer’s instructions. After transfer, the blot was blocked in Odyssey blocking buffer (927-40000, LI-COR Biosciences), and incubated overnight in Odyssey blocking buffer containing 0.1% (*v/v*) TWEEN and primary antibodies against AAC1 (ab102032, 1:500 (*v/v*) Abcam, Cambridge, UK), AAC2 (ab118125, 1:1000 (*v/v*), Abcam), AAC3 (ab154007, 1:2000 (*v/v*), Abcam) or COX5A (ab110262, 1:1000 (*v/v*), Abcam), as loading control at 4 °C, followed by incubation with corresponding secondary antibodies Alexa Fluor® 800 goat-anti-rabbit (926-32211, 1:10,000, LI-COR Biosciences) or Alexa Fluor® 680 goat-anti-mouse (926-68070 1:10,000, LI-COR Bioscience) in Odyssey blocking buffer containing 0.1% (*v/v*) TWEEN at room temperature for 1 h. Fluorescence was visualized using the Odyssey CLx scanner (Li-Cor Biosciences, USA).

### Functional ADP and ATP transport assessment

Cells were cultured and matured as described above. Mature cells were pelleted and resuspended in ice cold 10 mM Tris–HCl, pH 7.6, for isolation of mitochondrial enriched fractions. Cell suspension was mechanically homogenized (eight times) using a Potter–Elvehjem homogenizer on ice. To increase protein yield, a maximum of 3 million cells was homogenized at once. The homogenate was transferred to ice cold 1.5 M sucrose solution, mixed and centrifuged for 10 min at 2 °C and 600*g*. To obtain mitochondrial-enriched protein fractions, supernatant was spun down for 10 min at 2 °C and 14,000*g*, followed by resuspension of pellet in 10 mM Tris–HCl, pH 7.6 complemented with cOmplete™ Mini EDTA-free protease inhibitor, PhosSTOP™ and 0.1 mg/mL RNase-free DNase I (Roche, Basel, Switzerland). Mitochondrial protein in the isolated fraction was determined after three freeze/thaw cycles in liquid nitrogen, to break mitochondrial membranes, using Pierce™ BCA protein assay kit (Thermofisher Scientific) according to the manufacturer’s instructions. In order to evaluate AAC-dependent ADP import, 25 µg intact isolated mitochondria of wild type and *AAC3*^*−/−*^ ciPTEC were incubated in ADP import buffer, consisting of 250 mM sucrose, 20 mM HEPES, 10 mM KCl, 5 mM succinate, 3 mM KH_2_PO_4_, 1.5 mM MgCl_2_, 1 mM EGTA and 5 µM rotenone, pH 7.2, supplemented with 100 µM AAC inhibitor bongkrekic acid (BKA; B6179, Sigma-Aldrich), or MilliQ on ice in a multiscreen 96-wells HV filter plate (0.45 µM, MSHVN4B, Merck SKU). After 10 min, 1 µM [^14^C]-ADP (~ 1 µCi; # NEC559050UC, Perkin Elmer) was added for 15 min on ice, after which samples were vacuum aspirated and washed three times in ADP import buffer. Samples were resuspended in scintillation solvent (Perkin Elmer) and [^14^C]-ADP counts were quantified (Hidex 600SL, Turku, Finland). AAC-dependent ATP transport was measured by means of bioluminescence, using the ATP bioluminescence assay kit CLS II (#11699695001, Roche), according to the manufacturer’s instructions. In short, 25 µg intact isolated mitochondria were incubated with 100 µM BKA or MilliQ, for 10 min on ice. For the first measurement, ADP reaction buffer (250 mM sucrose, 20 mM HEPES, 10 mM KCl, 3 mM KH_2_PO_4_, 1.5 mM MgCl_2_, 1 mM EGTA, 1 mM malate and 1 mM pyruvate, pH 7.6 and kept at 28 °C) was added to a white/clear bottom 96-wells plate with 100 µM BKA or MilliQ and luciferase reagent (1:1, *v/v*). For the second measurement, pre-incubated mitochondria or MilliQ was added, followed by addition of 25 µM ADP in a third measurement. Bioluminescence signal was immediately measured for 0.1 s after each addition and 0.5 s shaking, on a Victor X3 multiplate reader (Perkin Elmer). Conditions without mitochondria, ADP or BKA served as control. All samples were analyzed at least in triplicate in three independent experiments.

### Measuring cellular metabolic activity

Effects of compounds on cellular metabolic activity were evaluated using the colorimetric tetrazolium salt-based MTT (3-(4,5-dimethylthiazol-2-yl)-2,5-diphenyl tetrazolium bromide) assay (Mosmann 1983). ciPTEC-OAT1 were seeded at a density of 63,000 cells/cm^2^ in transparent/clear flat bottom 96-wells plates, proliferated for 1 day at 33 °C and 5% (*v/v*) CO_2_ in PTEC complete medium, followed by 7 days maturation at 37 °C, 5% (*v/v*) CO_2_ to differentiate into an epithelial monolayer. Matured ciPTEC-OAT1 were exposed to known AAC inhibitors BKA, CATR, CD437 and suramin for 24 h at 37 °C and 5% (*v/v*) CO_2_. Relative drug concentrations applied were 0.01, 0.0316, 0.1, 0.316, 10, 31.6, 100, 316 and 1000 μM (dissolved in MilliQ or DMSO). Subsequently, cellular metabolic activity was assessed. In short, compound exposed cells were washed three times with serum-free PTEC complete medium (SFM) and incubated with 0.5 mg/mL MTT in SFM for 3 h at 37 °C and 5% (*v/v*) CO_2_. After dissolving formazan crystals in DMSO for 2 h on a microplate shaker, absorption was measured at 560 nm and subtracted from background at 670 nm using Benchmark (Bio-Rad, Veenendaal, The Netherlands). DMSO concentrations did not exceed 0.1% (*v/v*). Obtained results were normalized to vehicle (0.1% DMSO) exposed ciPTEC-OAT1.

### Cellular respiration and real-time ATP production rate measurements

To investigate cellular metabolism in both genetically and chemically inhibited cells, cellular oxygen consumption rates were measured using extracellular flux analyses (Seahorse XF96 Agilent, Santa Clara, CA, USA). 10,000 cells/well were seeded in 96-wells Seahorse XF96 cell culture microplates and matured as described above. Routinely culturing cells in high glucose conditions can inhibit mitochondrial function, known as the Crabtree effect, previously described for rapidly proliferating cancer cells (e.g., HepG2), thymocytes and human primary muscle cells (Aguer et al. 2011; Marroquin et al. 2007; Rossignol et al. 2004). Although a predominant oxidative phenotype for ciPTEC was previously suspected (Vriend et al. 2019), we attempted to stimulate mitochondrial respiration by replacing the glucose in the medium with galactose. While the glycolytic metabolism of glucose yields two net ATP molecules, pyruvate production via metabolism of galactose yields no ATP, and consequently increases reliance on oxidative phosphorylation for energy production (Marroquin et al. 2007). The effects of AAC interactors on mitochondrial respiration were evaluated in glucose-free DMEM (Sigma, D5030) complemented with 10 mM galactose, 0.64 mM MgCl_2_, 14.3 mM NaHCO_3_, 15 mM HEPES, 0.5 mM sodium pyruvate, and 2.5 mM glutamine, supplemented with ciPTEC growth factors mentioned above, pH 7.4 and 100 µM AAC inhibitors BKA, CATR, CD437, suramin or 0.1% DMSO for 12 h at 37 °C and 5% (*v/v*) CO_2_. Galactose-rich (10 mM) medium containing AAC inhibitors was replaced one hour before initiation of the experiment by NaHCO_3_-free DMEM containing 10 mM galactose, 0.5 mM sodium pyruvate, 31.7 mM NaCl, 5 mM HEPES and 2.5 mM glutamine, pH 7.4 and incubated at 37 °C without CO_2_. Oxygen consumption rate (OCR) and extracellular acidification rate (ECAR) were measured following subsequent addition of 2.5 µM oligomycin (Oli), 1 µM FCCP and 1 µM/2.5 µM rotenone (Rot)/antimycin A (AA). The effect of each addition was measured during 3 measurement cycles (each consisting of 3 min mixing and 3 min recording). For evaluation of ATP production rates, the buffer factor of the 10 mM galactose DMEM medium containing ciPTEC growth factors was empirically determined by titrating buffer pH with 5 mM HCl steps according to the manufacturer’s protocol. Non-mitochondrial respiration (after addition of Rot/AA) was subtracted from all OCR values. Basal (routine) OCR was determined in the absence of inhibitors at T = 14 min (point 3), while T = 54 min (i.e. in the presence of Oli and FCCP) was used as a measure of maximal respiration (point 9). Spare respiratory capacity was determined by subtracting routine OCR values from the maximal OCR values. Average data was obtained in N = 4 experiments (biological replicates), each consisting of four experimental replicates. Wave Desktop Software (Agilent) was used for data analysis. Measured OCR and ECAR rates were corrected for cell count. To this end, nuclei were stained with 20 µg/mL Hoechst 33,342 (Life Technologies) for 30 min at 37 °C, after which fluorescence was imaged using a 10 × objective Becton Dickinson (BD) Pathway 855 microscope (BD Bioscience, Breda, The Netherlands) using emission/excitation maxima of 380/435 nm.

### Citrate synthase activity

Mitochondrial mass was determined by citrate synthase activity in matured ciPTEC-OAT1 wild type and *AAC3*^*−/−*^, as previously described (Srere 1969). In short, cells were seeded and matured in transparent/clear flat bottom 96-wells plates as described above. Cells were lysed by 0.33% (*v/v*) Triton X-100 in 10 mM Tris–HCl, pH 7.6, followed by three freeze–thaw cycles in liquid nitrogen. Absorption was measured spectrophotometrically at 412 nm after addition of five volumes of 1 mM 5,5′-Dithiobis(2-nitrobenzoic acid) (DTNB, D8130, Sigma) with 10% (*v/v*) Triton X-100 in MilliQ, and 300 µM acetyl coenzyme A (A2181, Sigma), in a 25 °C prewarmed microplate reader (BioRad) using a kinetic protocol of medium mixing for 10 s with 20 s interval, followed by twenty cycles of 15 s interval without mixing. Total activity was measured after addition of 10 µL 10 mM oxaloacetic acid (A4126, Sigma) using the same kinetic protocol. Data analysis was performed in GraphPad Prism 9.0.0. For each measurement, slopes of at least five time points were defined, subtracted, and plotted.

### Mitochondrial morphology and function by TMRM

Cells were seeded and matured as described above in black/clear bottom flat 96-wells plates, after which mitochondrial morphology and membrane potential were assessed microscopically. Cells were washed once in HBSS, subsequently fluorescently stained with 25 nM tetramethylrhodamine methyl ester (TMRM) and co-stained with 0.75 µg/mL Hoechst 33342 in HBSS for 30 min at 37 °C, followed by image acquisition (40 × objective) and 12 × 12 montage using Becton Dickinson (BD) Pathway 855 microscope (BD Bioscience, Breda, The Netherlands), as described before (Iannetti et al. 2016). Acquired images were processed and quantified using Image-Pro Plus 6.0 software (Media Cybernetics, Rockville, MD, USA), as described previously (Iannetti et al. 2016). Average data was obtained in N = 3 experiments, consisting of 20 replicates each, and 8 cells/well were analyzed.

### Statistical analysis

Statistical data analysis was performed using GraphPad Prism 9.0.0 (GraphPad Software Inc., San Diego, CA). All data were normalized to wild type ciPTEC-OAT1 and presented as mean ± SEM of at least three independent experiments (N = 3), performed with five or six experimental replicates as indicated in the figure legends, unless stated otherwise. One-way ANOVA with Dunnett’s post hoc test or two-way ANOVA with Bonferroni post hoc test to correct for multiple comparison were used to evaluate differences between cell lines or responses, as indicated. Concentration-dependent effect of AAC inhibitors on cellular metabolic activity were fitted using nonlinear regression analysis.

## Results and discussion

### ***AAC3***^***−/−***^ knockout cells show a reduced ADP/ATP transporter activity and cellular oxidative metabolism

**Fig. 1:**
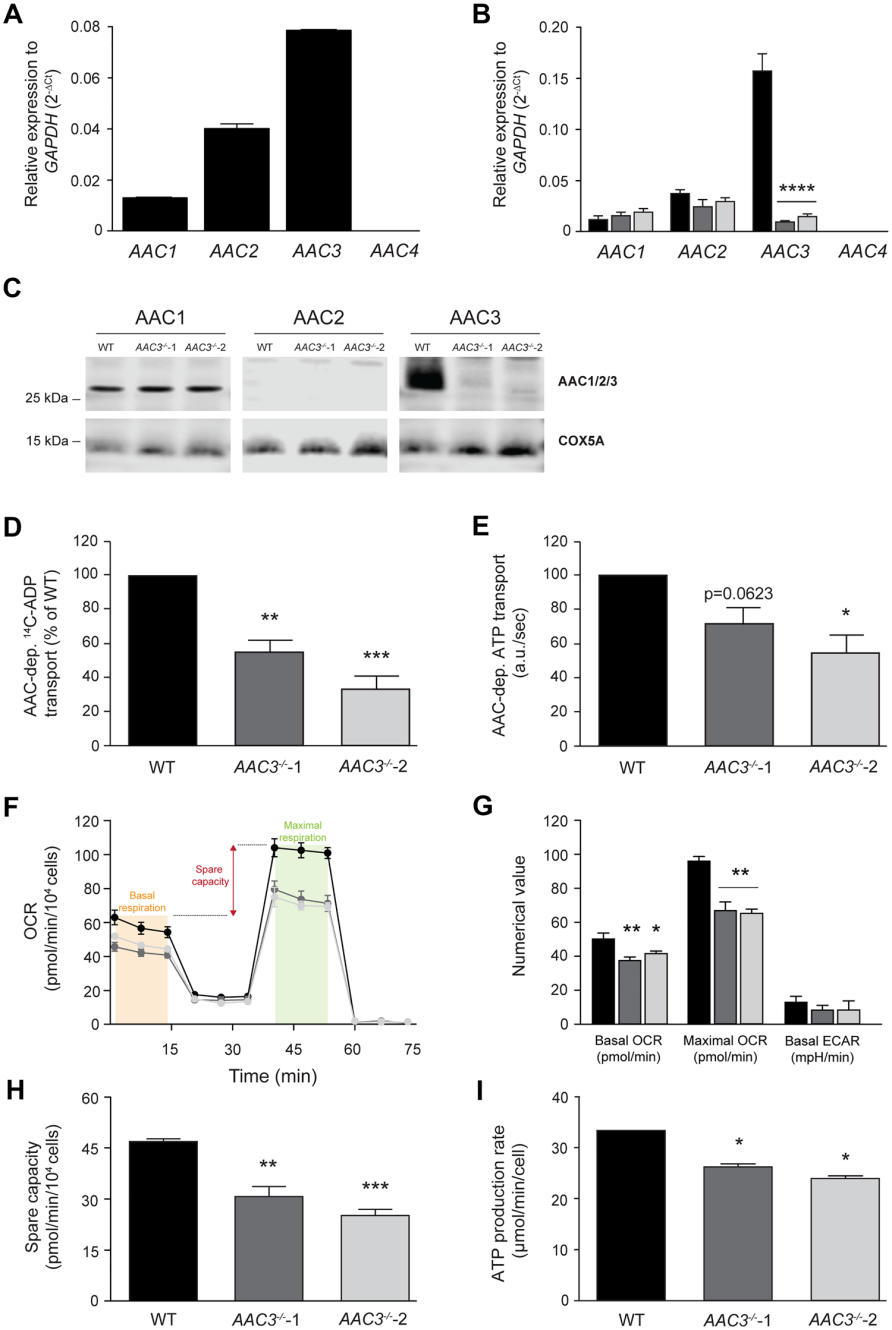
Development and validation of CRISPR/Cas9-mediated knockout of AAC3 in ciPTEC cells. Quantitative PCR was applied to assess the mRNA expression levels of the four human isoforms of AAC in conditionally immortalized proximal tubule epithelial cells (ciPTEC), before (**A**) and after (**B**) application of CRISPR/Cas9, for wild-type ciPTEC-OAT1 (black), *AAC3*^*−/−*^-1 (dark grey) and *AAC3*^*−/−*^-2 (light grey), relative to *GAPDH* expression. Two independently generated *AAC3* knockout cell lines were included in the experiments. In addition, protein levels of AAC1, AAC2 and AAC3 were determined by Western Blot (**C**) in which COX5A was used as loading control. AAC4 was excluded from analysis as qPCR revealed no mRNA expression, also in line with expression patterns described before (Dolce et al. 2005; Jang et al. 2008; Kunji et al. 2016b; Stepien et al. 1992). Functional AAC-mediated transport of ADP (**D**) and ATP (**E**) was investigated, using radioactivity and bioluminescence. Mitochondrial respiration was assessed by the mito stress test (Agilent), as described by the manufacturer, performed in culture medium containing 10 mM glucose, using the Seahorse XF Analyzer to evaluate oxygen consumption rates (OCR) and extracellular acidification rate (ECAR) of the different mitochondrial complexes (**F** and **G**), upon following injection of complex specific inhibitors oligomycin A (2.5 µM, complex V), carbonyl cyanide-*p*-trifluoromethoxyphenylhydrazone (FCCP, 1 µM) and rotenone/ antimycin A (1 µM/2.5 µM). Basal respiration (orange) consists of respiratory capacities under resting conditions, maximal respiration (green) is the maximal respiratory capacity of a cell. Spare capacity (red) was determined by subtracting basal respiration from maximal respiration (**F** and **H**). Cellular ATP production rate was assessed by the ATP rate assay, using the Seahorse XF Analyzer (**I**). Data was corrected for cell count, assessed by fluorescence microscopy after Hoechst staining. Significance was determined by one-way ANOVA with Dunnett’s post hoc analysis, *p < 0.05, **p < 0.01, ***p < 0.001 and ****p < 0.0001, mean ± SEM, N = 3 or 4 independent experiments

Human AAC has four different isoforms, for which expression patterns vary in a tissue- and disease-specific manner (Chevrollier et al. 2011; Chevrollier et al. 2005; Dolce et al. 2005; Jang et al. 2008; Kunji et al. 2016a; Stepien et al. 1992). Previously, the Human Protein Atlas Project identified AAC3 as most abundantly expressed isoform in renal cells. Similarly, we found AAC3 to be the predominant isoform in our renal ciPTEC cell model (Fig. 1A). Next, to better understand the function of AAC3 in cellular energy metabolism, the CRISPR/Cas9 approach was used to independently generate two *AAC3*^*−/−*^ proximal tubule epithelial cell lines, followed by genetic characterization of the knockout cell lines (Fig. 1 and Supplemental Fig. 1). First, quantitative PCR showed a significant reduction of AAC3-specific mRNA levels in both *AAC3*^*−/−*^ cell lines (Fig. 1B), whereas *AAC1* and *AAC2* were not affected. Sanger sequencing confirmed that targeting exon 1 resulted in two cell lines (*AAC3*^*−/−*^-1 and *AAC3*^*−/−*^-2, Supplemental Fig. 1) with severely shortened proteins of 61 and 60 amino acids, respectively, compared to 298 amino acids in wild type. Consequently, protein function was predicted to be disrupted. Protein expression was absent for AAC3 (~ 33 kDa), and unaffected for AAC1 (Fig. 1C), which suggests that no functional protein was properly folded. Remarkably, AAC2 could not be detected at ~ 33 kDa (Fig. 1C). However, a dimeric band (~ 66 kDa) was observed (Supplemental Fig. 2), which could be due to the detergent and its effect on the oligomeric state of membrane proteins in sample preparations, as discussed previously (Kunji and Ruprecht 2020). Next, we functionally characterized the *AAC3*^*−/−*^ and wild-type cell lines, to determine the impact of the truncated proteins. With respect to transporter activity, AAC-dependent ADP and ATP transport were assessed by measurement of direct import of ADP, using detection of radiolabeled ADP and an indirect method of ATP export using a bioluminescence assay, respectively. Both approaches demonstrated a reduced AAC-dependent ADP import and ATP export transporter activity, indicating that also on a functional level, gene editing did affect protein function (Fig. 1D and E). However, a substantial residual AAC activity was observed in *AAC3*^*−/−*^. To exclude that this residual transport was not due to the presence of fractionated mitochondria as a result of the preparation (isolation) procedure, this was evaluated by high-resolution respirometry using the Oxygraph-2 k, as previously published (Lanza and Nair 2009). The integrity of the outer mitochondrial membrane was not affected (Supplemental Fig. 4). Although the OCR remained high upon inhibition of ATP synthase (State 4 respiration), which represents the respiration required to maintain the mitochondrial membrane potential during uncoupling, an equivalent reduction in respiration rate from state 3 to state 4 was observed, at least for wild type and *AAC3*^*−/−*^ cells. This suggests that the lower ADP and ATP transport activity in *AAC3*^*−/−*^ (Fig. 1D and E) does not result from an increase in proton leakage in knockout cells compared to wild type. Alternatively, this observation could be explained by functional compensation through AAC1 and AAC2 (*i.e.* a higher transport rate), and not upregulation or increased protein expression, as mRNA and protein levels were not elevated for the different AAC isoforms (Fig. 1B**/**C and Supplemental Fig. 2). Unfortunately, isoform-specific alterations in AAC activity are difficult to assess, because of their highly similar structure, function and substrate selectivity (Kunji 2012; Kunji and Robinson 2006).

**Fig. 2:**
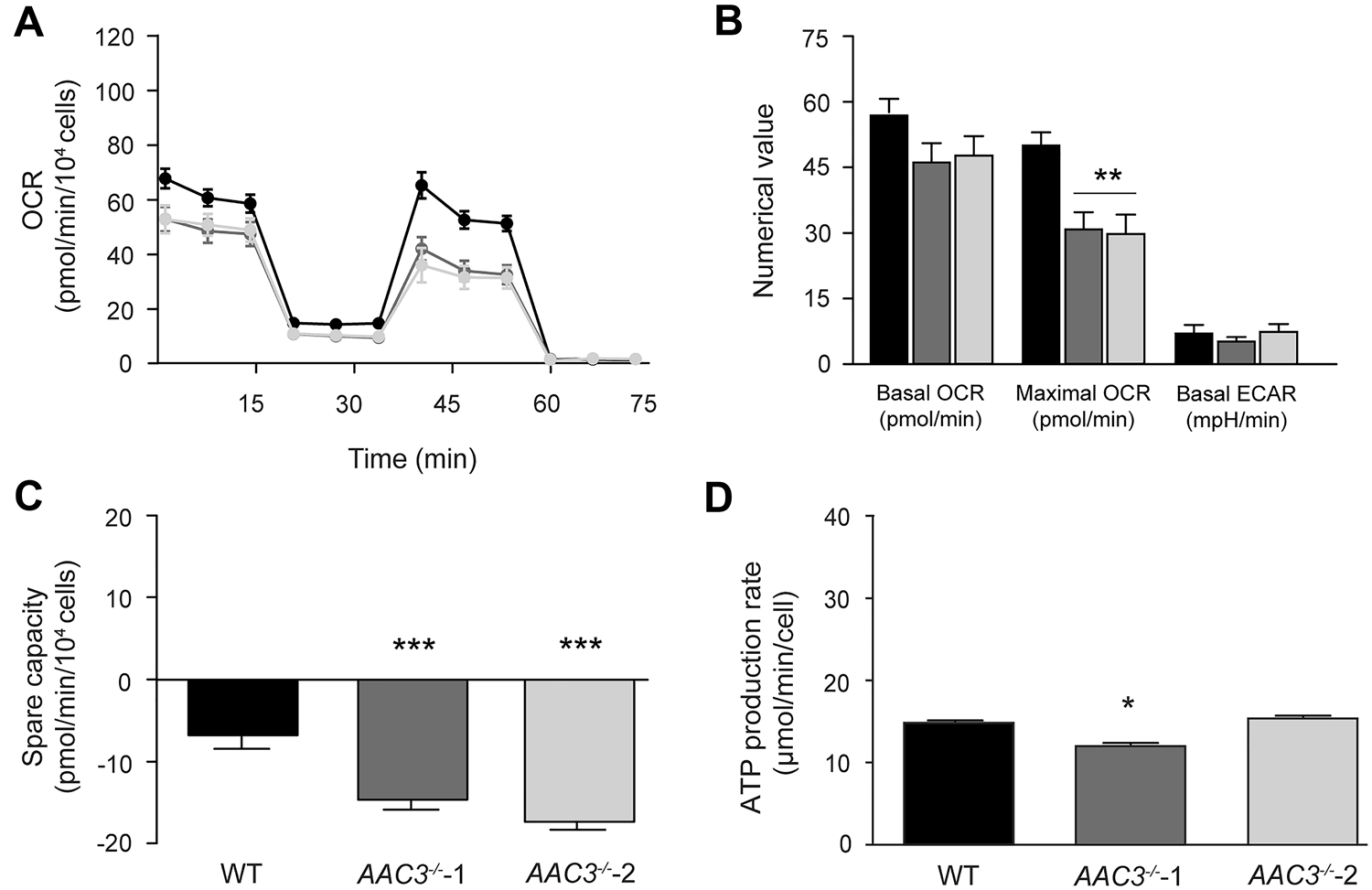
Stimulation of oxidative respiration by 10 mM galactose does not increase mitochondrial respiration in ciPTEC wildtype and* AAC3*^−/−^*.* The mito stress test assessed key parameters of mitochondrial function by measuring the oxygen consumption rate (OCR) and extracellular acidification rate (ECAR) upon consecutive injection of oligomycin, FCCP and a combined injection of rotenone and antimycin, using Seahorse XF Analyzer (**A** and **B**). Spare capacity was determined by subtracting basal from maximal respiration rates (**C**). Cellular ATP production was assessed by measuring the ATP rate using the Seahorse XF Analyzer (**D**). Data was corrected for cell count, assessed by fluorescence microscopy after Hoechst staining. Significance was determined by one-way ANOVA with Dunnett’s post hoc analysis, *p < 0.05, **p < 0.01 and ***p < 0.001, mean ± SEM, N = 3 independent experiments

To further evaluate the effects of *AAC3*^*−/−*^ on mitochondrial and cellular metabolism, we investigated oxygen consumption and ATP production rates in wild-type and *AAC3*^*−/−*^ cells. Compared to wild type, both *AAC3*^*−/−*^ cell lines showed a significant decrease in basal and maximal respiration (OCR), while low basal ECAR levels did not differ between cell lines (Fig. 1F, G and Supplemental Fig. 3A), suggesting that cells mainly use OXPHOS-mediated ATP generation and do not compensate for the absence of *AAC3* by increased glycolytic activity. Spare respiratory capacity was reduced in the *AAC3*^*−/−*^ cells (Fig. 1H). Moreover, ATP production rates were significantly lower in *AAC3*^*−/−*^ cells compared to wild type, suggesting mitochondrial dysfunction (Fig. 1I). To further probe the effects on oxidative metabolism, we used galactose as an energy source instead of glucose in an attempt to stimulate mitochondrial metabolism (Fig. 2) (Aguer et al. 2011). Under these conditions, maximal OCR was reduced compared to basal levels, indicating that under non-stressed conditions (i.e., basal respiration) cells used their complete oxidative respiratory capacity and were not able to adapt under stressed (i.e., maximal respiration) conditions (Fig. 2A and B). Remarkably, the basal OCR of these cells in galactose was similar compared to glucose conditions, suggestive of an overall oxidative phenotype of these cells independent of the energy source. It was indeed previously shown that ciPTEC cells overexpressing the organic anion transporter 1 (ciPTEC-OAT1) are characterized by reduced extracellular lactate levels, while intracellular ATP levels were retained, which was attributed to the shift towards an oxidative phenotype in these cells (Vriend et al. 2019). This is in accordance with our observation that basal ECAR was low in both glucose- and galactose-conditioned media (Fig. 2B and Supplemental Fig. 3). The low glycolytic capacity may be explained by the metabolic nature of renal proximal tubular cells, which has been described to mainly depend on fatty acid β-oxidation (Simon and Hertig 2015). However, this is only indirect evidence, as a correct interpretation of ECAR is challenging due to confounding interference by other sources of extracellular acidification, such as the reduction of exogenous pyruvate to lactate (Schmidt et al. 2021). Further research is required to understand why respiration rates remained unchanged in these cells under galactose conditions.

The importance of the spare capacity used under these conditions is shown by the fact that under uncoupled conditions (i.e., maximal respiration) both *AAC3*^*−/−*^ cell lines fail to thrive because respiration collapses even below basal rates (Fig. 2A–C). In line with this finding, ATP production rate was significantly reduced in *AAC3*^*−/−*^-1 (Fig. 2D).

### ***AAC3***^***−/−***^ affects mitochondrial mass but not their morphology

**Fig. 3:**
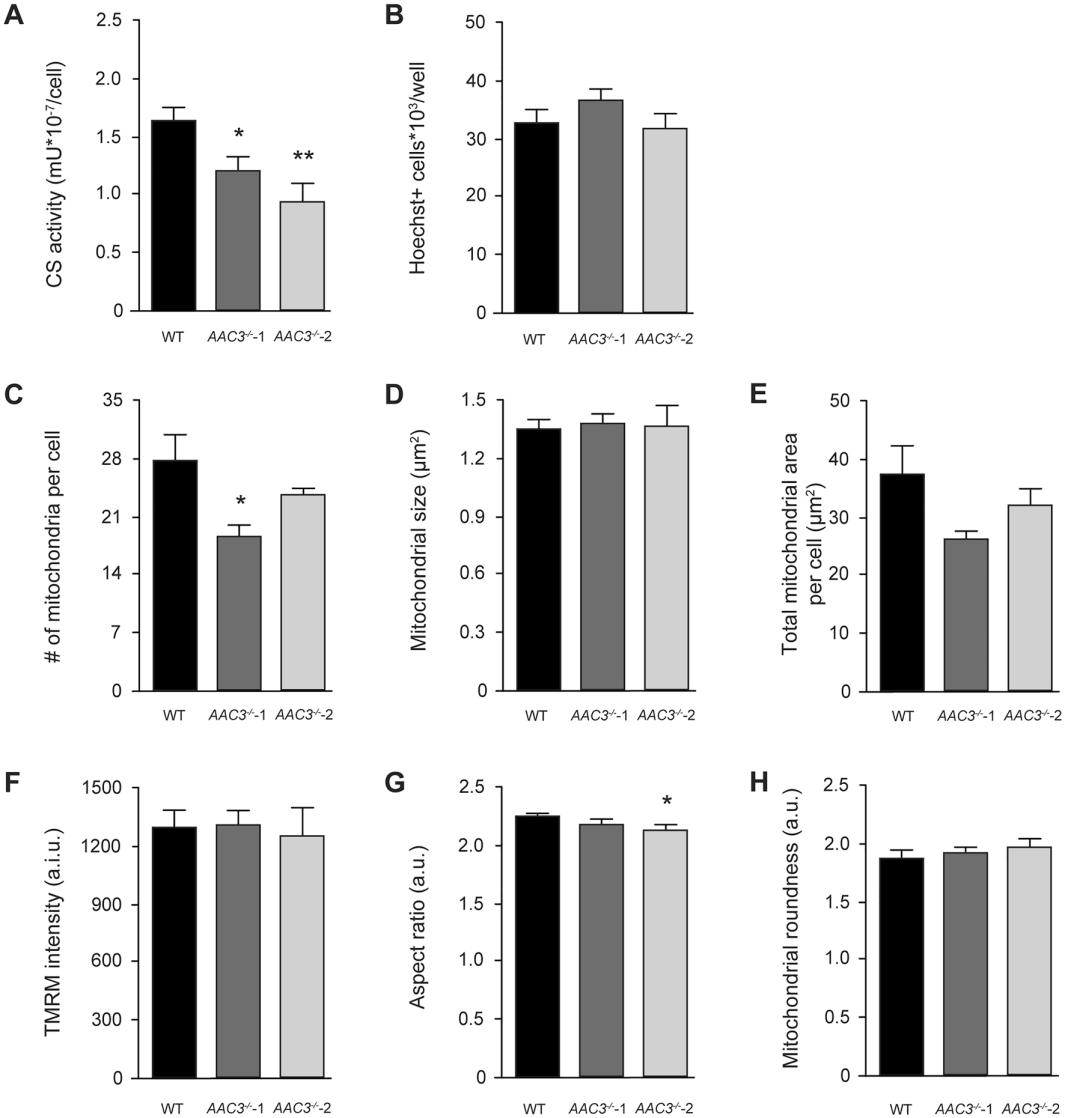
Quantitative assessment of mitochondrial morphology and function in ciPTEC-OAT1 and *AAC3*^*−/−*^*.* ciPTEC-OAT1 (black), *AAC3*^*−/−*^-1 (dark grey) and *AAC3*^*−/−*^-2 (light grey) were matured and evaluated on a functional and morphological level (**A**). Citrate synthase (CS) activity was determined (**B**). In addition, cells were evaluated by fluorescence microscopic assessment using 25 nM tetramethylrhodamine methyl ester (TMRM), as described before (Iannetti et al. 2016). Co-staining with Hoechst was performed to correct for cell count. The number of mitochondria per cell (**C**), mitochondrial size (**D**), total mitochondrial area per cell (**E**), mitochondrial membrane potential (**F**), aspect ratio (a measure of mitochondrial length: ratio between major and minor axis) (**G**) and mitochondrial roundness (measure of mitochondrial length and degree of branching) (**H**) were evaluated. Significance was determined by one-way ANOVA with Dunnett’s post hoc analysis, *p < 0.05 and **p < 0.01, mean ± SEM, N = 3 independent experiments

AAC deficiency has previously been associated with an increased mitochondrial mass (*i.e.*, citrate synthase activity) (Palmieri et al. 2005) and, therefore, we investigated mitochondrial content in our *AAC3*^*−/−*^ cell lines. Remarkably, we found that citrate synthase (CS) activity per cell was significantly reduced in both *AAC3*^−/−^, while the total number of cells was unaffected (Fig. 3A and B). To estimate whether mitochondrial mass was indeed decreased in the AAC3 knockout cell lines, we measured the number of mitochondrial objects per cell using TMRM staining and fluorescence microscopy. The number of mitochondria per cell was significantly reduced in *AAC3*^*−*/−^-1 and appeared lower in *AAC3*^*−*/−^-2 (Fig. 3C). Quantification of mitochondrial size (Fig. 3D) allowed calculation of the total mitochondrial area per cell, which was not significantly reduced in the AAC knockout cell lines (Fig. 3E). This may suggest that the observed decrease in mitochondrial mass measured by CS activity was relatively small. It also supports the idea that the observed residual ADP/ATP exchange activity is indeed explained by functional compensation of the other AAC isoforms, described above. Analysis of mitochondrial TMRM staining intensity revealed no differences between cell lines (Fig. 3F), whereas mitochondrial aspect ratio was slightly reduced in *AAC3*^*−*/−^-2 cells (Fig. 3G). No difference in mitochondrial formfactor was observed (Fig. 3H). This suggests that the mitochondrial membrane potential is maintained by a mechanism that is independent of AAC3 or OCR, which requires further investigation.

### Oxidative conditions increase susceptibility to AAC inhibition

**Fig. 4:**
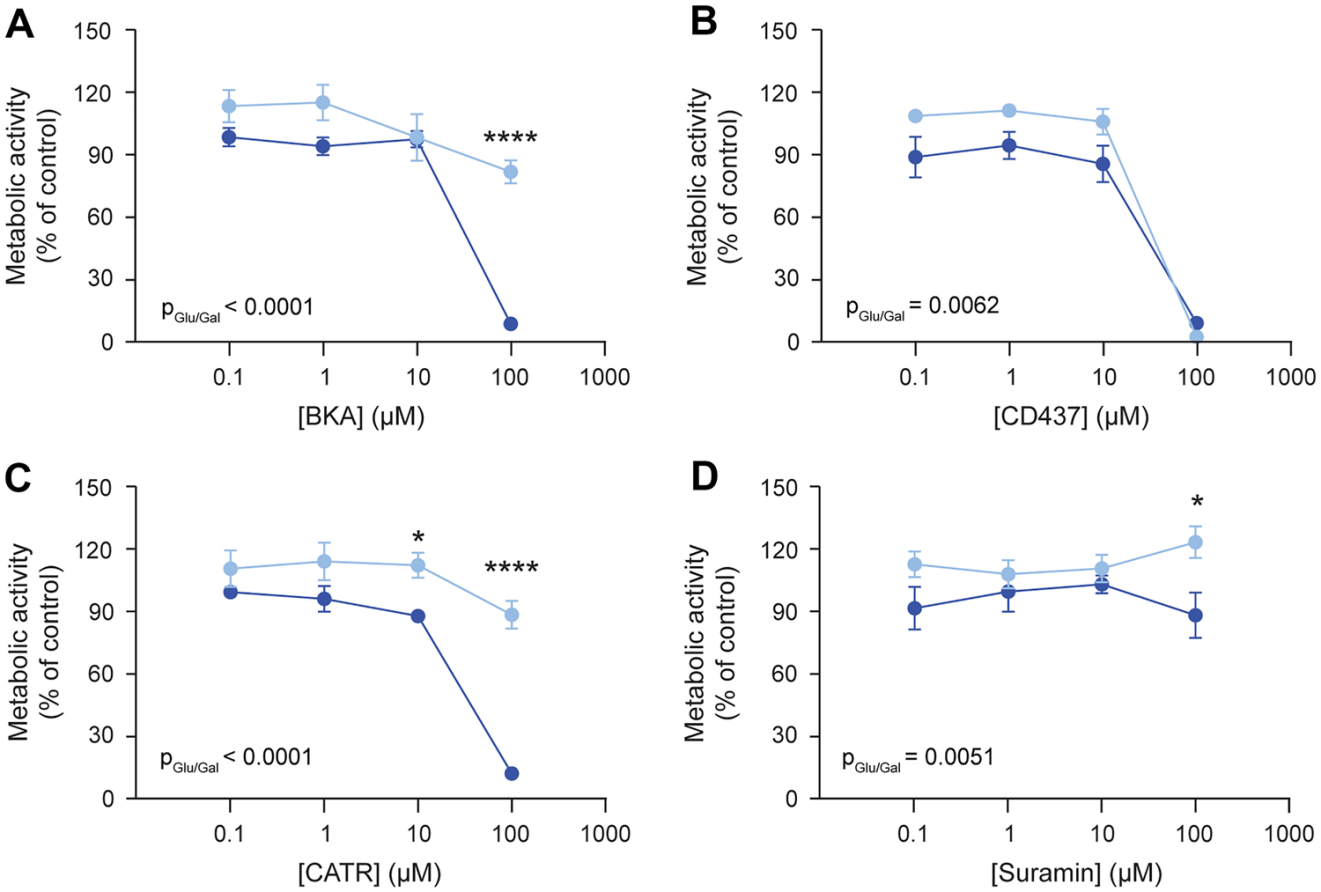
Concentration-dependent effects of AAC inhibitors on cellular metabolic activity in ciPTEC wild type using the glucose / galactose assay. Wild-type ciPTEC-OAT1 cells were matured and exposed to increasing concentrations of AAC inhibitors bongkrekic acid (BKA, **A**), CD437 (**B**), carboxyatractyloside (CATR, **C**), suramin (**D**) or 0.1% DMSO for 24 h in medium containing 10 mM glucose (light blue) or galactose (dark blue) at 37 °C and 5% (*v/v*) CO_2_, followed by measurement of cellular metabolic activity by means of MTT. All results were normalized to DMSO vehicle controls. Statistical analyses: two-way ANOVA corrected for multiple comparison by Bonferroni’s post hoc analysis to compare differences in metabolic activity between medium composition conditions. p value corresponding to overall significance between media composition is indicated for each compound (p_Glu/Gal_), *p < 0.05 and ****p < 0.0001. Mean ± SEM; N = 3 independent experiments

As described above, the AAC3 knockout cell lines have residual ADP/ATP exchange and respiratory capacity, which may point towards functional compensation by other AAC isoforms. Consequently, we aimed to investigate the effects of AAC inhibitors on cellular metabolism in wild type and *AAC3*^*−/−*^*.* Under standard glucose conditions, cell-permeable AAC inhibitors BKA and CD437 reduced the metabolic activity measured by MTT (Fig. 4A and B) (Jaiquel Baron et al. 2021). Interestingly, switching the carbon source to galactose, making these cells solely reliant on mitochondrial oxidative capacity, enhanced metabolic inhibition. CATR, the most potent AAC inhibitor (Vignais et al. 1966), also showed a significant concentration-dependent reduction in cellular metabolic activity in wild-type cells (Fig. 4C). The previously reported AAC inhibitor suramin (Jaiquel Baron et al. 2021), did not cause a significant reduction in metabolic capacity (Fig. 4D), but this may have been due to its poor cell membrane permeability caused by its high molecular weight (1.297 Da) and large negatively charged surface areas (Herzig et al. 2012). Cells cultured in medium containing galactose showed a reduced metabolic capacity compared to ‘standard’ culture medium containing glucose (Fig. 4A–D, p-values between assay are indicated). The observed significant reduction in cellular metabolic activity at 10 μM CATR may relate to the strong inhibitory capacity of CATR on AAC activity, reducing ADP import from the cytosol into the mitochondrial matrix. As a result, OXPHOS complex V is not active and the ATP remaining in the mitochondrial matrix may initiate reverse electron transfer (RET). Consequently, succinate dehydrogenase (complex II) could operate in the reverse mode. As MTT is a substrate of (mitochondrial) dehydrogenase activity, it may explain its reduced conversion. However, additional data supporting this hypothesis is lacking.

**Fig. 5:**
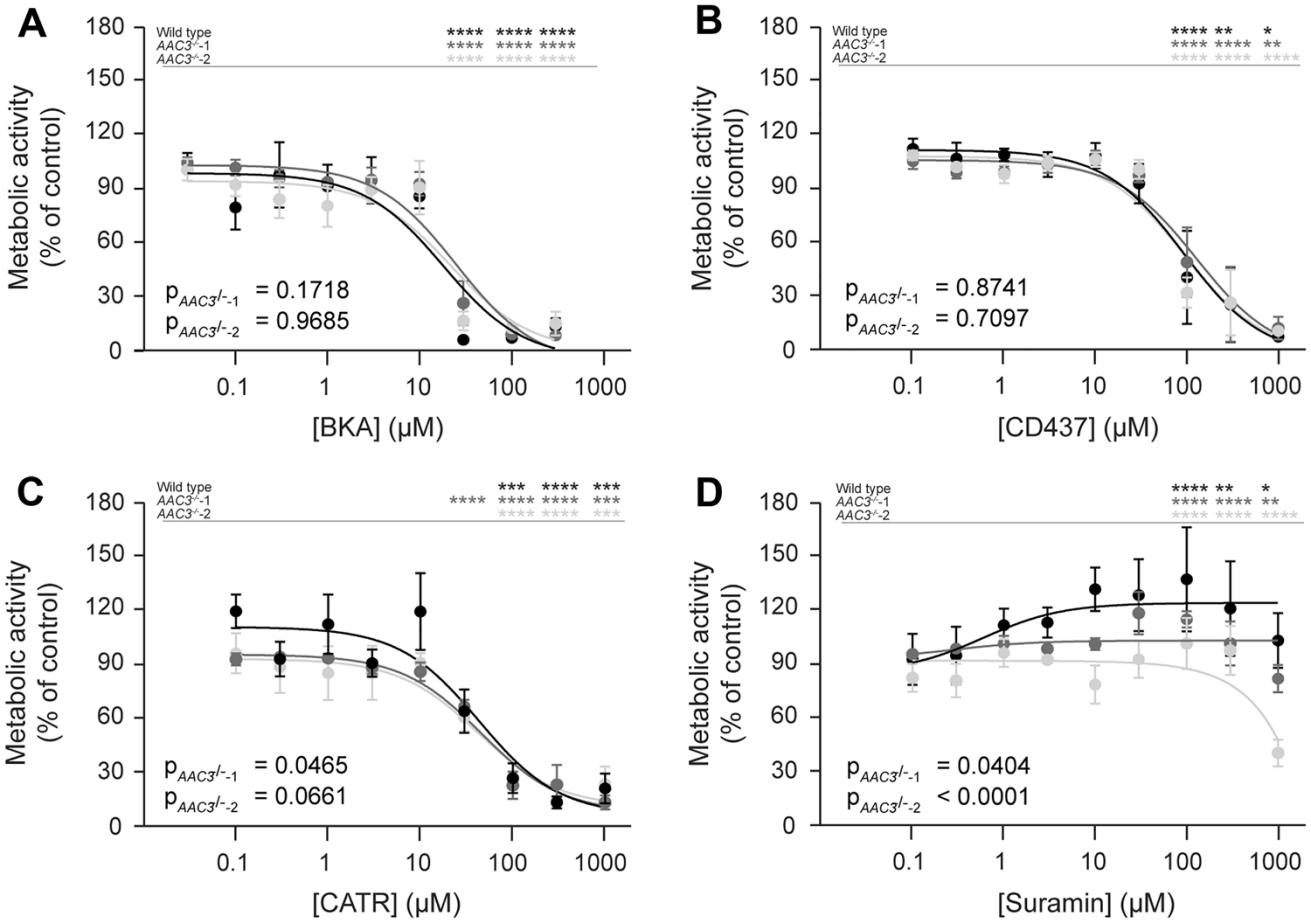
Concentration-dependent effects of AAC inhibitors on cellular metabolic activity in ciPTEC wild type and *AAC3*^*−/−*^*.* Mature wild-type ciPTEC-OAT1 cells (black), *AAC3*^*−/−*^-1 (dark grey) and *AAC3*^*−/−*^-2 (light grey) were cultured, matured and exposed to increasing concentrations of AAC inhibitors bongkrekic acid (BKA, **A**), CD437 (**B**), carboxyatractyloside (CATR, **C**), suramin (**D**) or 0.1% DMSO for 24 h in medium containing 10 mM galactose, followed by measurement of cellular metabolic activity by means of MTT. All results were normalized to DMSO vehicle controls. Statistical analyses: one-way ANOVA corrected for multiple comparison by Dunnett’s post hoc analysis to compare differences between vehicle control and exposed conditions within a cell line: *p < 0.05, **p < 0.01, ***p < 0.001 and ****p < 0.0001, indicated in the upper table of each graph) and two-way ANOVA corrected for multiple comparison by Bonferroni’s post hoc analysis to compare overall significant differences between knockout cell line and wild type concentration-dependent responses, as indicated (p_AAC3_^−/−^_-1_ or p_AAC3_^−/−^_-2_). Mean ± SEM; N = 3 independent experiments

Since the effects of AAC inhibition were most pronounced in galactose medium, we further investigated the dose-dependent effects of all four AAC interactors in wild type and both *AAC3*^*−/−*^ ciPTEC clones (Fig. 5A–D). Our hypothesis was that AAC3 knockout cells would be less sensitive compared to wild type as they lack the pharmacological target (i.e., AAC). Comparison of wild-type cells with *AAC3*^*−/−*^only revealed a statistical difference for CATR in *AAC3*^*−/−*^-1. The lack of differences between knockouts and wild type suggests again that the other AAC isoforms could compensate for a possible decreased sensitivity.

### Genetic and chemical AAC inhibition reduce mitochondrial respiration

**Fig. 6:**
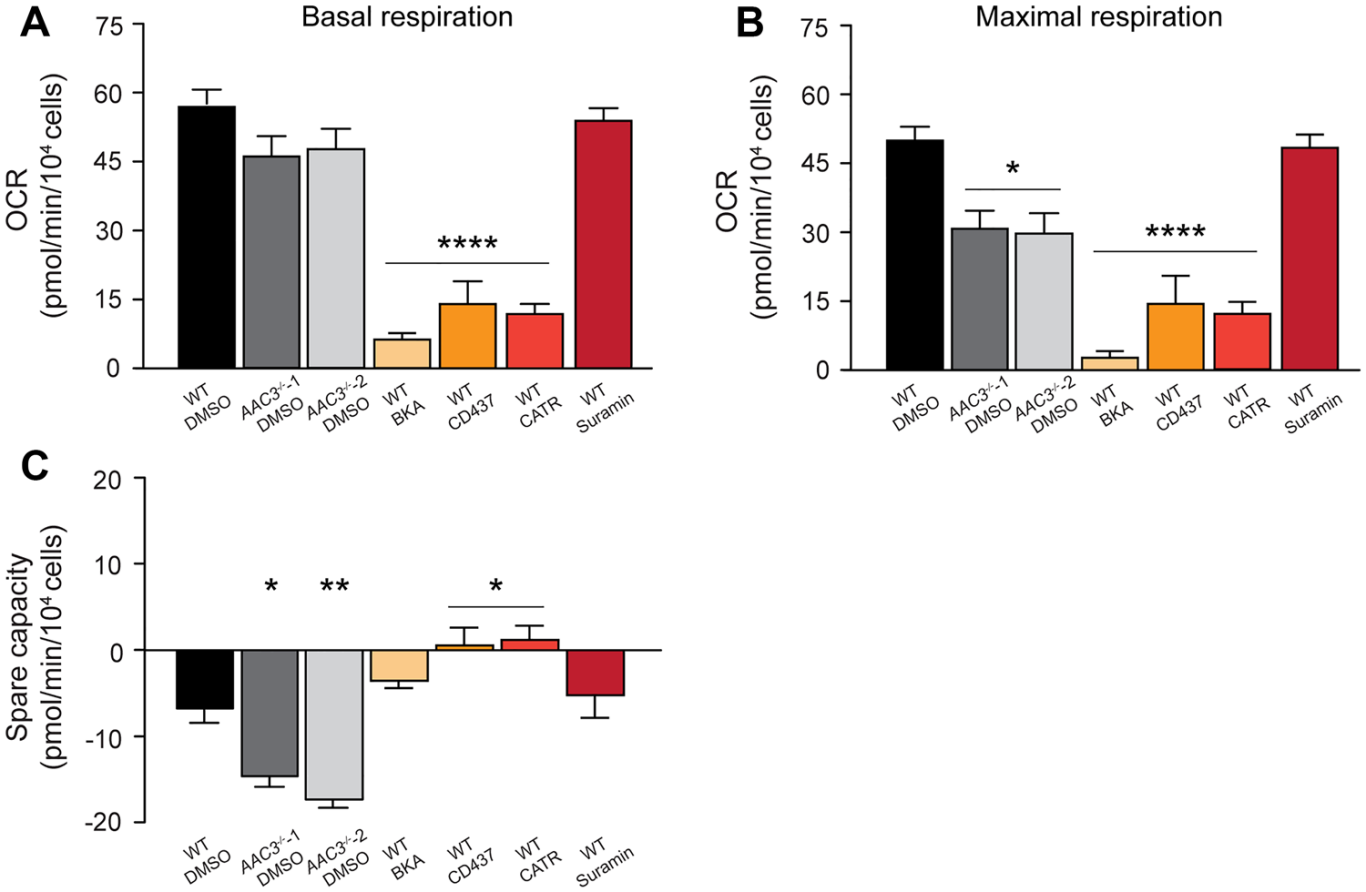
Genetic and chemical AAC3 inhibition reduce mitochondrial respiration. Mature wild-type ciPTEC-OAT1 cells (black and orange/red) were exposed to 0.1% DMSO (black) or 100 µM AAC inhibitors BKA (light orange), CD437 (middle orange), CATR (dark orange) and suramin (red) medium containing 10 mM galactose at 37 °C and 5% (*v/v*) CO_2_. Generated AAC3 knockouts (*AAC3*^*−/−*^-1, dark grey and *AAC3*^*−/−*^-2, light grey) were incubated with 0.1% DMSO in medium containing 10 mM galactose at 37 °C and 5% (*v/v*) CO_2_. After 12 h, mitochondrial respiration was evaluated using the Seahorse XF Analyzer. Oxygen consumption rates for all mitochondrial complexes were investigated. Data shown represents basal (**A**) and maximal respiration (**B**) and spare capacity (**C**). Data was corrected for cell count, assessed by fluorescence microscopy after Hoechst staining. Significance was determined by one-way ANOVA with Dunnett’s post hoc analysis, *p < 0.05, **p < 0.01, ***p < 0.001 and ****p < 0.0001, mean ± SEM, N = 3 independent experiments, comparing all conditions to wild type (black). Results of basal and maximal respiration and spare capacity shown in panels **A–C** for wildtype (black) and *AAC3*^*−/−*^ (dark and light grey) are also presented in Fig. 2

To characterize the *AAC3*^*−/−*^ cells in more detail, we examined whether they recapitulated chemical inhibition of AAC, in particular the effects on mitochondrial respiration, as observed in Fig. 1F–H and Fig. 2A–C. Wild-type and *AAC3*^*−/−*^ ciPTEC were exposed to AAC inhibitors in 10 mM galactose medium to boost oxidative metabolism, followed by evaluation of mitochondrial respiratory capacity using the Seahorse XF Analyzer. All compounds, except for suramin, significantly reduced basal and maximal OCR in wild-type cells, compared to vehicle controls (Fig. 6A and B). Interestingly, chemical AAC inhibition showed a significantly stronger reduction in OCR compared to the two vehicle-exposed *AAC3*^*−/−*^ cell lines (p < 0.05), supporting the idea that the residual mitochondrial respiration observed in our genetically removed AAC3 model may be explained by functional compensation of the remaining AAC isoforms, as described above.

This is further supported by the fact that mitochondrial transport carriers, including AAC, are known for their highly conserved residues in the substrate binding site and the large sequence homology (Bhatia et al. 2020; Kunji and Robinson 2006; Pebay-Peyroula et al. 2003; Robinson et al. 2008; Ruprecht et al. 2014, 2019). In addition, comparison of the isoforms demonstrated only minor differences in IC50-values for CD437 (Zhang et al. 2016). Therefore, chemical inhibition is not expected to be selective for one isoform, resulting in equal reduction of AAC1, 2, 3, and 4 functions. Our genetic knockout model was designed to specifically target the most abundant isoform AAC3, which could result in functional compensation by AAC1, 2 and 4, as observed in our study. Next, we calculated the resulting respiratory spare capacity, for which a subtle, but stronger effect was observed in *AAC3*^*−/−*^ compared to chemically-inhibited wild-type cells (Fig. 6C). However, under all conditions there was no significant spare capacity when ETC was maximally stimulated compared to basal respiration levels, suggesting that metabolic compensation to restore cell function is impaired under energy-demanding conditions (viz*.* galactose and not glucose as carbon source). Moreover, specific deletion of AAC3 does not increase the sensitivity of cells for AAC inhibitors (Supplemental Fig. 5). Recapitulating the exact similar effect of chemical AAC inhibition in a genetic model might be challenging. Although in yeast a quadruple knockout has resulted in a viable cell (Hatanaka et al. 1999), it is expected to be technically difficult to generate a full knockout of all human isoforms.

## Conclusions

In this study, we show that ciPTEC-OAT1 cells predominantly depend on oxidative metabolism for cellular function and that replacement of the energy source (viz*.* galactose instead of glucose) does not stimulate mitochondrial respiration. In addition, AAC is involved in maintaining mitochondrial OCR, as both basal and maximal OCR decrease upon genetic and chemical inhibition of AAC3. Particularly under energy demanding conditions, this may have consequences for cellular function. Chemical inhibition of AAC by established inhibitors reduces basal and maximal respiration even stronger compared to genetically inhibited AAC3, suggesting a role for functional AAC compensation in our *AAC3*^*−/−*^ model.

## Supplementary Information

Below is the link to the electronic supplementary material.

Supplemental Figure 1 | Validation of genetic sequence of *AAC3*^-/-^ in ciPTEC-OAT1 using DNA sanger sequencing in two independently generated knockout cell lines, *AAC3*^-/-^-1 and *AAC3*^-/-^-2, and wildtype. Distinct deletions were identified by aligning the sequences of *AAC3*^-/-^ ciPTEC to wild-type ciPTEC using SnapGene Viewer and Clustal Omega, and mapping on the homo sapiens reference genome GCRh38.p13. CRISPR/Cas9-mediated gene editing resulted in frameshift mutations, leading to premature stop codons and shortened protein for AAC3^-/-^-1 and AAC3^-/-^-2 cell lines of 161 and 160 amino acids, respectively, in comparison to 298 amino acids for wild-type AAC3. (TIF 27853 KB)

Supplemental Figure 2 | Full western blots of AAC1, 2 and 3 expression in wild-type and *AAC3*^-/-^ cells. Primary antibodies: AAC1 (ab102032, 1:500 Abcam), AAC2 (ab118125, 1:1000, Abcam), AAC3 (ab154007, 1:2000, Abcam) and COX5A (ab110262, 1:1000, Abcam), as loading control. Secondary antibodies: Alexa Fluor® 800 goat-anti-rabbit (926-32211, 1:10000, LI-COR Biosciences) or Alexa Fluor® 680 goat-anti-mouse (926-68070 1:10000, LI-COR Biosciences. Predicted band sizes for AAC1, 2 and 3 is 33 kDa and 17 kDa for COX5A. However, observed band size (according to the manufacturer) for COX5A is 13 kDa, which is in line with our observations. Arrow indicates predicted dimer formation of AAC2, as previously observed, potentially due to contribution of the detergent in sample preparation, as extensively reviewed by Kunji et al.(Kunji and Ruprecht 2020). Aspecific fragments were present. (TIF 28160 KB)

Supplemental Figure 3 | Evaluation of extracellular acidification rates (ECAR) in wild type (black) and the two generated *AAC3*^-/-^ (dark and light grey) ciPTEC upon consecutive injection of oligomycin, FCCP and antimycin A + rotenone, at time points indicated, using the Seahorse XF Analyzer. Cells were cultured for 12h in glucose (10 mM, A) or galactose (10 mM, B) medium, after which ECAR was determined. (TIF 26410 KB)

Supplemental Figure 4 | Evaluating mitochondrial membrane integrity in isolated wild type and *AAC3*^-/-^ mitochondria-enriched fractions by high-resolution respirometry. To ensure that mitochondrial membrane integrity was not lost due to sample preparation, wild type (black) and *AAC3*^-/-^ (dark and light grey) ciPTEC were matured and mitochondria-enriched fractions were obtained by homogenizing cells and differential centrifugation, as described. Mitochondrial oxidative capacity and integrity of the mitochondrial membrane of these isolated fractions were evaluated using the high-resolution Oxygraph-2k respirometer, provided with Datlab 5 recording and analysis software (Oroboros Instruments, Innsbruck, Austria). A previously published protocol was followed to specifically determine mitochondrial membrane integrity after the isolation procedure (Lanza and Nair 2009). In two thermostated chambers (37°C), isolated mitochondria were added for each cell line, followed by evaluation of substrate-specific effects on cellular respiration. In short, baseline mitochondrial respiration represented the consumption of oxygen in absence of exogenous substrates. Addition of glutamate (10 mM, # G1251, Sigma-Aldrich) and malate (2mM, # M1000, Sigma-Aldrich) reflected oxygen consumption at OXPHOS complex I (State 2). Subsequent injections of saturating ADP levels (4 mM total, # A4386, Sigma-Aldrich) maximally stimulated complex I-driven respiration (State 3). Cytochrome C (10 μM, # C2506, Sigma-Aldrich) was then injected as a quality control to confirm the integrity of the outer mitochondrial membrane (OMM), followed by succinate (10 mM, # S2378, Sigma-Aldrich) to also stimulate complex II (State 3, CI+II) and rotenone (0.5 μM, # R8875, Sigma-Aldrich) to inhibit complex I and to measure complex II-driven respiration (State 3, CII). Lastly, oligomycin (2.5 μM, # O4876, Sigma-Aldrich) was added to inhibit ATP synthase and determine a proton leak across the inner mitochondrial membrane (State 4). A representative oxygen flux rate Oxygraph-2k plot for wild type cells (Suppl. Fig. 4A) and background-corrected analysis of oxygen flux rate are shown (Suppl. Fig. 4B). All data was corrected to the amount of mitochondrial protein added. Respiration rates were determined by the average value of oxygen flux prior to subsequent injection. Although the data presented resulted from one experiment, all measurements were performed in duplicate for each cell line, for which mitochondria were independently isolated. The preparation procedure did not affect the outer mitochondrial membrane (OMM integrity). N=1 experiment, measured in duplicate for each cell line. Mitochondria were obtained in three isolation rounds, one for each cell type. (TIF 26664 KB)

Supplemental Figure 5 | Genetic AAC3 inhibition does not increase sensitivity of cells for AAC inhibitors. Mature wild-type ciPEC-OAT1 cells (black), and generated AAC3 knockouts (*AAC3*^-/-^-1, dark grey and *AAC3*^-/-^-2, light grey) were exposed to 100 µM AAC inhibitors BKA (A and B), CD437 (C and D), CATR (E and F), suramin (G and H) or 0.1% DMSO in medium containing 10 mM galactose for 12 hours at 37°C and 5% (v/v) CO2, followed by evaluation of mitochondrial respiration using the Seahorse XF Analyzer. Oxygen consumption rates (OCR) for all mitochondrial complexes were investigated. Data shown represents basal and maximal respiration (left) and spare capacity (right). Data was corrected for cell count, assessed by fluorescence microscopy after Hoechst staining. Significance was determined by one-way ANOVA with Dunnett’s post hoc analysis, *p<0.05, **p<0.01, ***p<0.001 and ****p<0.0001, mean ± SEM, N=3 independent experiments. Wild-type data is also presented in figure 5. (TIF 26793 KB)

## Data Availability

The data generated and analyzed during the current study are available from the corresponding authors on reasonable request.

## References

[CR1] Aguer C, Gambarotta D, Mailloux RJ (2011). Galactose enhances oxidative metabolism and reveals mitochondrial dysfunction in human primary muscle cells. PLoS ONE.

[CR2] Bhatia D, Capili A, Choi ME (2020). Mitochondrial dysfunction in kidney injury, inflammation, and disease: potential therapeutic approaches. Kidney Res Clin Pract.

[CR3] Chevrollier A, Loiseau D, Stepien G (2005). What is the specific role of ANT2 in cancer cells?. Med Sci (paris).

[CR4] Chevrollier A, Loiseau D, Reynier P, Stepien G (2011). Adenine nucleotide translocase 2 is a key mitochondrial protein in cancer metabolism. Bba-Bioenergetics.

[CR5] Dolce V, Scarcia P, Iacopetta D, Palmieri F (2005). A fourth ADP/ATP carrier isoform in man: identification, bacterial expression, functional characterization and tissue distribution. FEBS Lett.

[CR6] Gai Z, Gui T, Kullak-Ublick GA, Li Y, Visentin M (2020). The role of mitochondria in drug-induced kidney injury. Front Physiol.

[CR7] Gutierrez-Aguilar M, Baines CP (2013). Physiological and pathological roles of mitochondrial SLC25 carriers. Biochem J.

[CR8] Haitina T, Lindblom J, Renstrom T, Fredriksson R (2006). Fourteen novel human members of mitochondrial solute carrier family 25 (SLC25) widely expressed in the central nervous system. Genomics.

[CR9] Hatanaka T, Hashimoto M, Majima E, Shinohara Y, Terada H (1999). Functional expression of the tandem-repeated homodimer of the mitochondrial ADP/ATP carrier in Saccharomyces cerevisiae. Biochem Biophys Res Commun.

[CR10] Heidari R (2019). The footprints of mitochondrial impairment and cellular energy crisis in the pathogenesis of xenobiotics-induced nephrotoxicity, serum electrolytes imbalance, and Fanconi's syndrome: a comprehensive review. Toxicology.

[CR11] Herzig S, Raemy E, Montessuit S (2012). Identification and functional expression of the mitochondrial pyruvate carrier. Science (new York, NY).

[CR12] Hoste EA, Bagshaw SM, Bellomo R (2015). Epidemiology of acute kidney injury in critically ill patients: the multinational AKI-EPI study. Intensive Care Med.

[CR13] Huls M, Brown CD, Windass AS (2008). The breast cancer resistance protein transporter ABCG2 is expressed in the human kidney proximal tubule apical membrane. Kidney Int.

[CR14] Iannetti EF, Smeitink JAM, Beyrath J, Willems PHGM, Koopman WJH (2016). Multiplexed high-content analysis of mitochondrial morphofunction using live-cell microscopy. Nat Protoc.

[CR15] Ishimoto Y, Inagi R (2016). Mitochondria: a therapeutic target in acute kidney injury. Nephrol Dial Transplant.

[CR16] Jaiquel Baron S, King MS, Kunji ERS, Schirris TJJ (2021). Characterization of drug-induced human mitochondrial ADP/ATP carrier inhibition. Theranostics.

[CR17] Jang JY, Choi Y, Jeon YK, Aung KCY, Kim CW (2008). Over-expression of adenine nucleotide translocase 1 (ANT1) induces apoptosis and tumor regression in vivo. BMC Cancer.

[CR18] Klingenberg M (2008). The ADP and ATP transport in mitochondria and its carrier. Biochem Biophys Acta.

[CR19] Koepsell H (2013). The SLC22 family with transporters of organic cations, anions and zwitterions. Mol Aspects Med.

[CR20] Kunji ERS (2011) Structural and mechanistic aspects of mitochondrial transport proteins. In: Comprehensive Biophysics, vol 8: bioenergetics, pp 174–205 10.1016/B978-0-12-374920-8.00814-6

[CR21] Kunji ER, Ferguson SA (2012). Structural and mechanistic aspects of mitochondrial transport proteins. Comprehensive biophysics.

[CR22] Kunji ERS, Robinson AJ (2006). The conserved substrate binding site of mitochondrial carriers. BBA-Bioenergetics.

[CR23] Kunji ERS, Ruprecht JJ (2020). The mitochondrial ADP/ATP carrier exists and functions as a monomer. Biochem Soc Trans.

[CR24] Kunji ER, Aleksandrova A, King MS (2016). The transport mechanism of the mitochondrial ADP/ATP carrier. Biochem Biophys Acta.

[CR25] Kunji ERS, Aleksandrova A, King MS (2016). The transport mechanism of the mitochondrial ADP/ATP carrier. BBA-Mol Cell Res.

[CR26] Kunji ERS, King MS, Ruprecht JJ, Thangaratnarajah C (2020). The SLC25 carrier family: important transport proteins in mitochondrial physiology and pathology. Physiology (bethesda).

[CR27] Lanza IR, Nair KS (2009). Functional assessment of isolated mitochondria in vitro. Methods Enzymol.

[CR28] Launay-Vacher V, Izzedine H, Karie S, Hulot JS, Baumelou A, Deray G (2006). Renal tubular drug transporters. Nephron Physiol.

[CR29] Li Y, Couch L, Higuchi M, Fang JL, Guo L (2012). Mitochondrial dysfunction induced by sertraline, an antidepressant agent. Toxicol Sci.

[CR30] Lill R, Kispal G (2001). Mitochondrial ABC transporters. Res Microbiol.

[CR31] Luciani S, Martini N, Santi R (1971). Effects of carboxyatractyloside a structural analogue of atractyloside on mitochondrial oxidative phosphorylation. Life Sci II.

[CR32] Manuel MA, Weiner MW (1977). Effects of ethacrynic acid and furosemide on phosphorylation reactions of kidney mitochondria. Inhibition of the adenine nucleotide translocase. Biochem Biophys Acta.

[CR33] Marroquin LD, Hynes J, Dykens JA, Jamieson JD, Will Y (2007). Circumventing the Crabtree effect: replacing media glucose with galactose increases susceptibility of HepG2 cells to mitochondrial toxicants. Toxicol Sci.

[CR34] Mehta RL, Pascual MT, Soroko S (2004). Spectrum of acute renal failure in the intensive care unit: the PICARD experience. Kidney Int.

[CR35] Moffett BS, Goldstein SL (2011). Acute kidney injury and increasing nephrotoxic-medication exposure in noncritically-ill children. Clin J Am Soc Nephrol.

[CR36] Moreno-Sanchez R, Bravo C, Vasquez C, Ayala G, Silveira LH, Martinez-Lavin M (1999). Inhibition and uncoupling of oxidative phosphorylation by nonsteroidal anti-inflammatory drugs: study in mitochondria, submitochondrial particles, cells, and whole heart. Biochem Pharmacol.

[CR37] Mosmann T (1983). Rapid colorimetric assay for cellular growth and survival: application to proliferation and cytotoxicity assays. J Immunol Methods.

[CR38] Motohashi H, Inui K (2013). Organic cation transporter OCTs (SLC22) and MATEs (SLC47) in the human kidney. AAPS J.

[CR39] Nieskens TT, Peters JG, Schreurs MJ (2016). A human renal proximal tubule cell line with stable organic anion transporter 1 and 3 expression predictive for antiviral-induced toxicity. AAPS J.

[CR40] Palevsky PM, Liu KD, Brophy PD (2013). KDOQI US commentary on the 2012 KDIGO clinical practice guideline for acute kidney injury. Am J Kidney Dis.

[CR41] Palmieri F (2004). The mitochondrial transporter family (SLC25): physiological and pathological implications. Pflugers Arch.

[CR42] Palmieri F, Monne M (2016). Discoveries, metabolic roles and diseases of mitochondrial carriers: a review. Biochim Biophys Acta.

[CR43] Palmieri F, Pierri CL (2010). Mitochondrial metabolite transport. Essays Biochem.

[CR44] Palmieri L, Alberio S, Pisano I (2005). Complete loss-of-function of the heart/muscle-specific adenine nucleotide translocator is associated with mitochondrial myopathy and cardiomyopathy. Hum Mol Genet.

[CR45] Palmieri F, Scarcia P, Monne M (2020). Diseases caused by mutations in mitochondrial carrier genes SLC25: a review. Biomolecules.

[CR46] Pebay-Peyroula E, Dahout-Gonzalez C, Kahn R, Trezeguet V, Lauquin GJM, Brandolin R (2003). Structure of mitochondrial ADP/ATP carrier in complex with carboxyatractyloside. Nature.

[CR47] Perazella MA (2018). Pharmacology behind common drug nephrotoxicities. Clin J Am Soc Nephrol.

[CR48] Prasad B, Johnson K, Billington S (2016). Abundance of drug transporters in the human kidney cortex as quantified by quantitative targeted proteomics. Drug Metab Dispos.

[CR49] Robinson AJ, Overy C, Kunji ERS (2008). The mechanism of transport by mitochondrial carriers based on analysis of symmetry. Proc Natl Acad Sci USA.

[CR50] Rossignol R, Gilkerson R, Aggeler R, Yamagata K, Remington SJ, Capaldi RA (2004). Energy substrate modulates mitochondrial structure and oxidative capacity in cancer cells. Cancer Res.

[CR51] Ruprecht JJ, Kunji ER (2019). Structural changes in the transport cycle of the mitochondrial ADP/ATP carrier. Curr Opin Struct Biol.

[CR52] Ruprecht JJ, Kunji ERS (2020). The SLC25 mitochondrial carrier family: structure and mechanism. Trends Biochem Sci.

[CR53] Ruprecht JJ, Hellawell AM, Harding M, Crichton PG, McCoy AJ, Kunji ER (2014). Structures of yeast mitochondrial ADP/ATP carriers support a domain-based alternating-access transport mechanism. Proc Natl Acad Sci USA.

[CR54] Ruprecht JJ, King MS, Zogg T (2019). The molecular mechanism of transport by the mitochondrial ADP/ATP carrier. Cell.

[CR55] Salet C, Moreno G, Morliere P, Santus R (1991). Effects of anthralin on mitochondrial bioenergetics. Arch Dermatol Res.

[CR56] Schirris TJJ, Ritschel T, Renkema GH, Willems PHGM, Smeitink JAM, Russel FGM (2015). Mitochondrial ADP/ATP exchange inhibition: a novel off-target mechanism underlying ibipinabant-induced myotoxicity. Sci Rep.

[CR57] Schmidt CA, Fisher-Wellman KH, Neufer PD (2021). From OCR and ECAR to energy: perspectives on the design and interpretation of bioenergetics studies. J Biol Chem.

[CR58] Siew ED, Davenport A (2015). The growth of acute kidney injury: a rising tide or just closer attention to detail?. Kidney Int.

[CR59] Simon N, Hertig A (2015). Alteration of fatty acid oxidation in tubular epithelial cells: from acute kidney injury to renal fibrogenesis. Front Med (lausanne).

[CR60] Smeets PH, van Aubel RA, Wouterse AC, van den Heuvel JJ, Russel FG (2004). Contribution of multidrug resistance protein 2 (MRP2/ABCC2) to the renal excretion of p-aminohippurate (PAH) and identification of MRP4 (ABCC4) as a novel PAH transporter. J Am Soc Nephrol.

[CR61] Soltoff SP (1986). ATP and the regulation of renal cell function. Annu Rev Physiol.

[CR62] Srere PA (1969). Citrate synthase: [EC 4.1.3.7. Citrate oxaloacetate-lyase (CoA-acetylating)]. Methods Enzymol.

[CR63] Stepien G, Torroni A, Chung AB, Hodge JA, Wallace DC (1992). Differential expression of adenine nucleotide translocator isoforms in mammalian tissues and during muscle cell differentiation. J Biol Chem.

[CR64] Todisco S, Di Noia MA, Onofrio A (2016). Identification of new highly selective inhibitors of the human ADP/ATP carriers by molecular docking and in vitro transport assays. Biochem Pharmacol.

[CR65] Uchino S, Kellum JA, Bellomo R (2005). Acute renal failure in critically ill patients: a multinational, multicenter study. JAMA.

[CR66] van der Meer V, Wielders HP, Grootendorst DC (2010). Chronic kidney disease in patients with diabetes mellitus type 2 or hypertension in general practice. Br J Gen Pract.

[CR67] Vignais PV, Duee ED, Vignais PM, Huet J (1966). Effects of atractyligenin and its structural analogues on oxidative phosphorylation and on the translocation of adenine nucleotides in mitochondria. Biochem Biophys Acta.

[CR68] Vriend J, Hoogstraten CA, Venrooij KR (2019). Organic anion transporters 1 and 3 influence cellular energy metabolism in renal proximal tubule cells. Biol Chem.

[CR69] Wilmer MJ, Saleem MA, Masereeuw R (2010). Novel conditionally immortalized human proximal tubule cell line expressing functional influx and efflux transporters. Cell Tissue Res.

[CR70] Zhang YJ, Tian DF, Matsuyama H (2016). Human adenine nucleotide translocase (ANT) modulators identified by high-throughput screening of transgenic yeast. J Biomol Screen.

