# Supplementary figures and images for: Metabolic impact of genetic and chemical ADP/ATP carrier inhibition in renal proximal tubule epithelial cells
Source: Arch Toxicol. 2023 May 8;97(7):1927–41. doi: 10.1007/s00204-023-03510-7 (PMC10256673; doi:10.1007/s00204-023-03510-7)

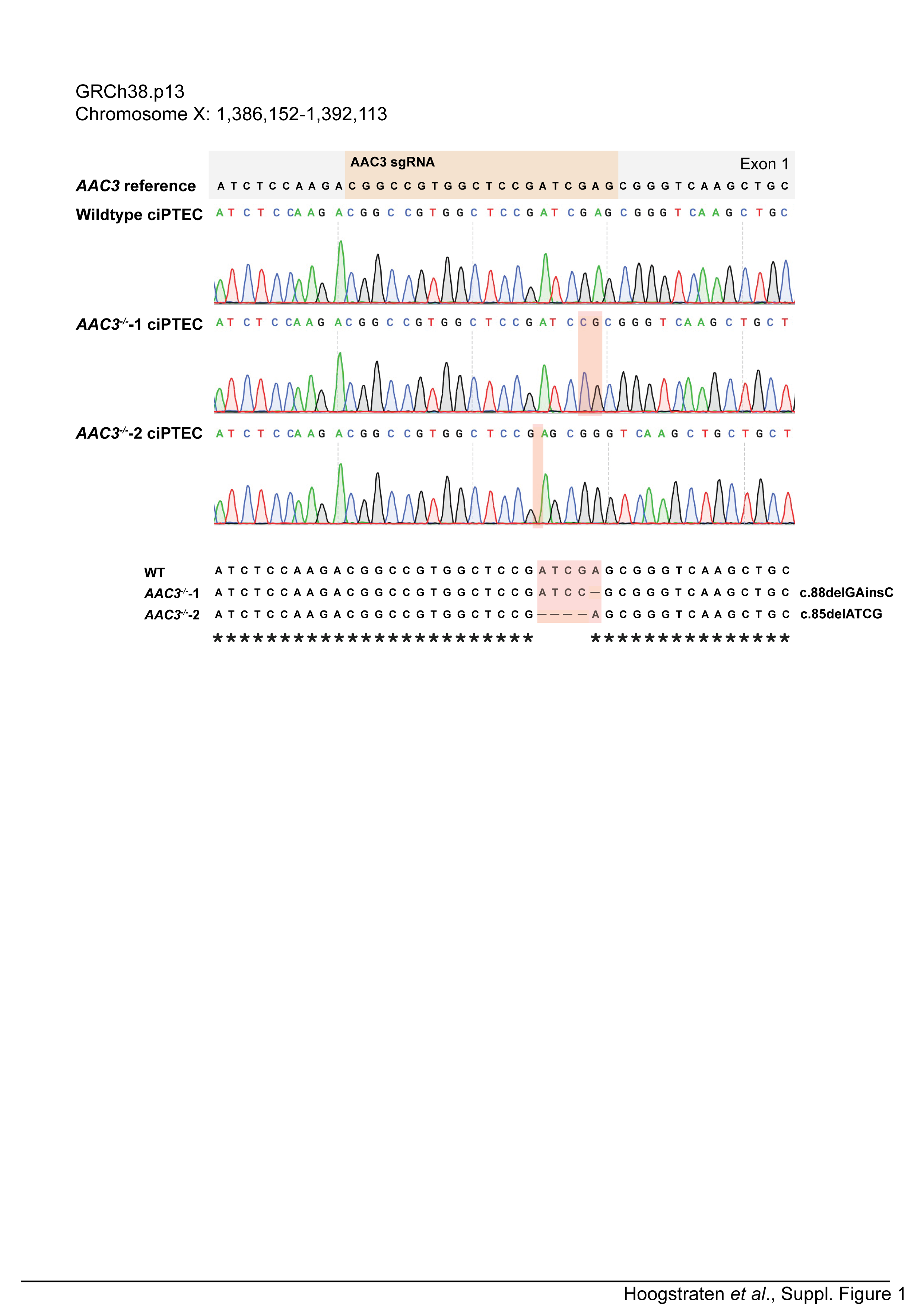

Supplement: Supplementary file 1 — Supplemental Figure 1 | Validation of genetic sequence of AAC3-/- in ciPTEC-OAT1 using DNA sanger sequencing in two independently generated knockout cell lines, AAC3-/--1 and AAC3-/--2, and wildtype. Distinct deletions were identified by aligning the sequences of AAC3-/- ciPTEC to wild-type ciPTEC using SnapGene Viewer and Clustal Omega, and mapping on the homo sapiens reference genome GCRh38.p13. CRISPR/Cas9-mediated gene editing resulted in frameshift mutations, leading to premature stop codons and shortened protein for AAC3-/--1 and AAC3-/--2 cell lines of 161 and 160 amino acids, respectively, in comparison to 298 amino acids for wild-type AAC3. (TIF 27853 KB) [file 204_2023_3510_MOESM1_ESM.tif]

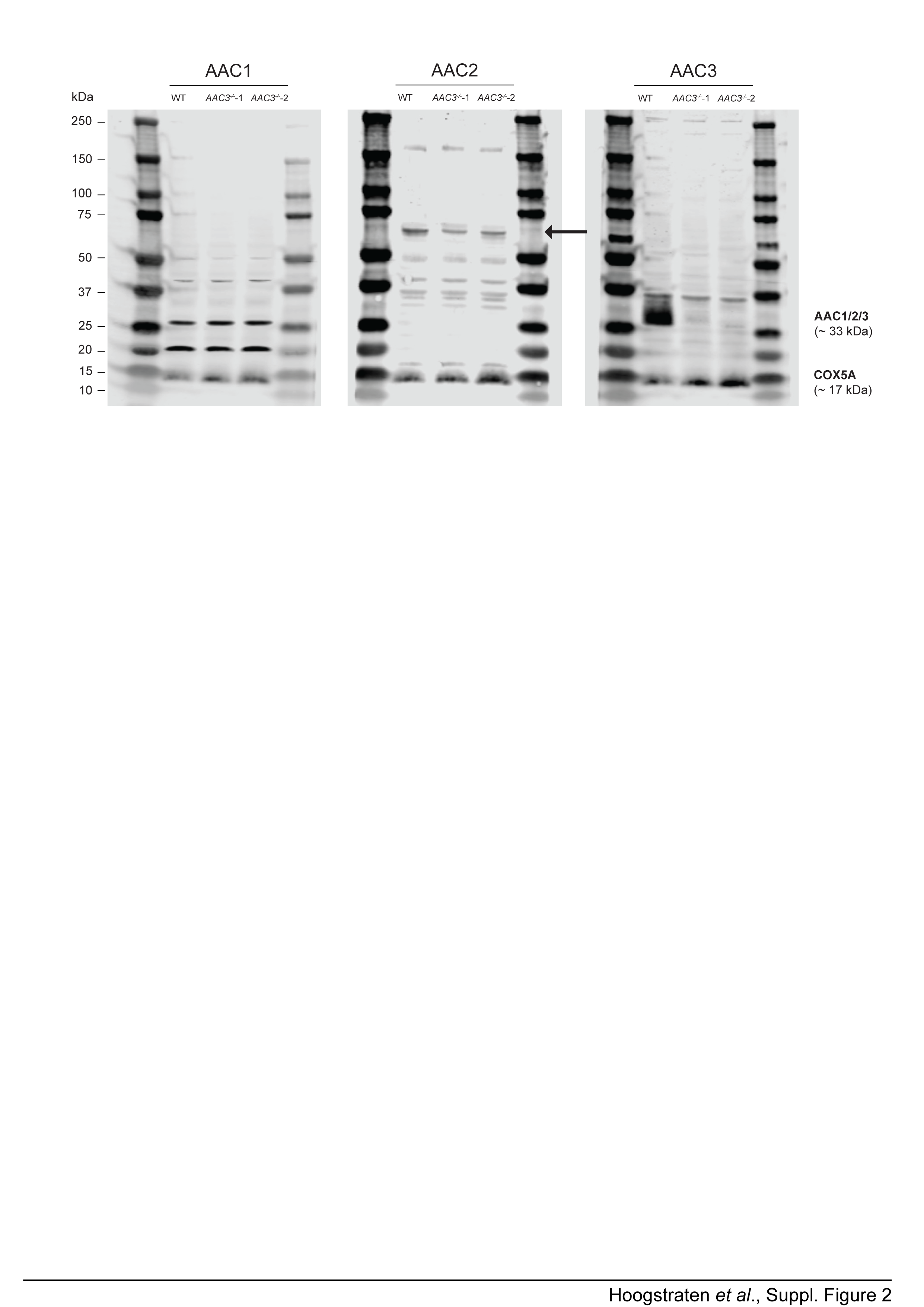

Supplement: Supplementary file 2 — Supplemental Figure 2 | Full western blots of AAC1, 2 and 3 expression in wild-type and AAC3-/- cells. Primary antibodies: AAC1 (ab102032, 1:500 Abcam), AAC2 (ab118125, 1:1000, Abcam), AAC3 (ab154007, 1:2000, Abcam) and COX5A (ab110262, 1:1000, Abcam), as loading control. Secondary antibodies: Alexa Fluor® 800 goat-anti-rabbit (926-32211, 1:10000, LI-COR Biosciences) or Alexa Fluor® 680 goat-anti-mouse (926-68070 1:10000, LI-COR Biosciences. Predicted band sizes for AAC1, 2 and 3 is 33 kDa and 17 kDa for COX5A. However, observed band size (according to the manufacturer) for COX5A is 13 kDa, which is in line with our observations. Arrow indicates predicted dimer formation of AAC2, as previously observed, potentially due to contribution of the detergent in sample preparation, as extensively reviewed by Kunji et al.(Kunji and Ruprecht 2020). Aspecific fragments were present. (TIF 28160 KB) [file 204_2023_3510_MOESM2_ESM.tif]

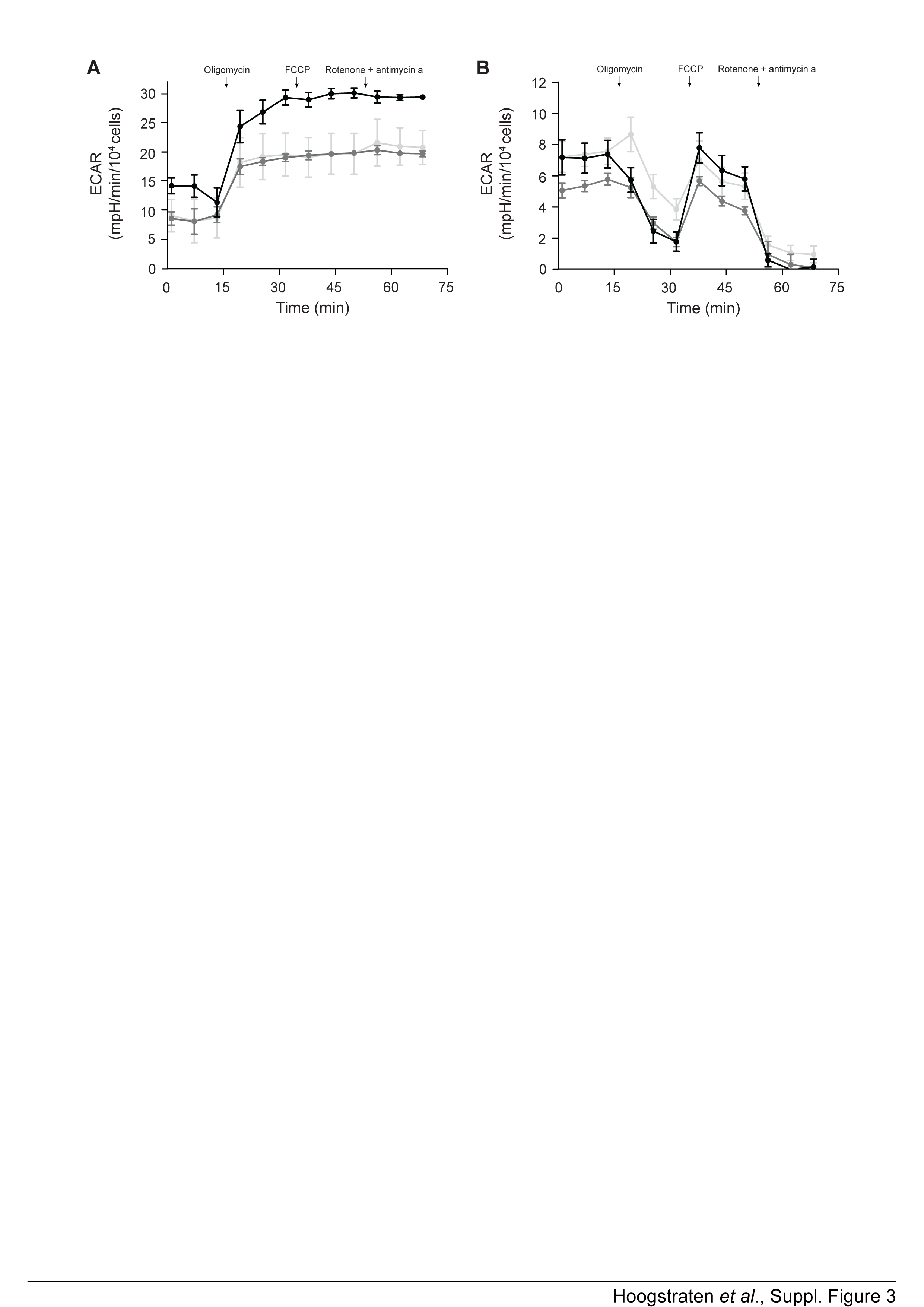

Supplement: Supplementary file 3 — Supplemental Figure 3 | Evaluation of extracellular acidification rates (ECAR) in wild type (black) and the two generated AAC3-/- (dark and light grey) ciPTEC upon consecutive injection of oligomycin, FCCP and antimycin A + rotenone, at time points indicated, using the Seahorse XF Analyzer. Cells were cultured for 12h in glucose (10 mM, A) or galactose (10 mM, B) medium, after which ECAR was determined. (TIF 26410 KB) [file 204_2023_3510_MOESM3_ESM.tif]

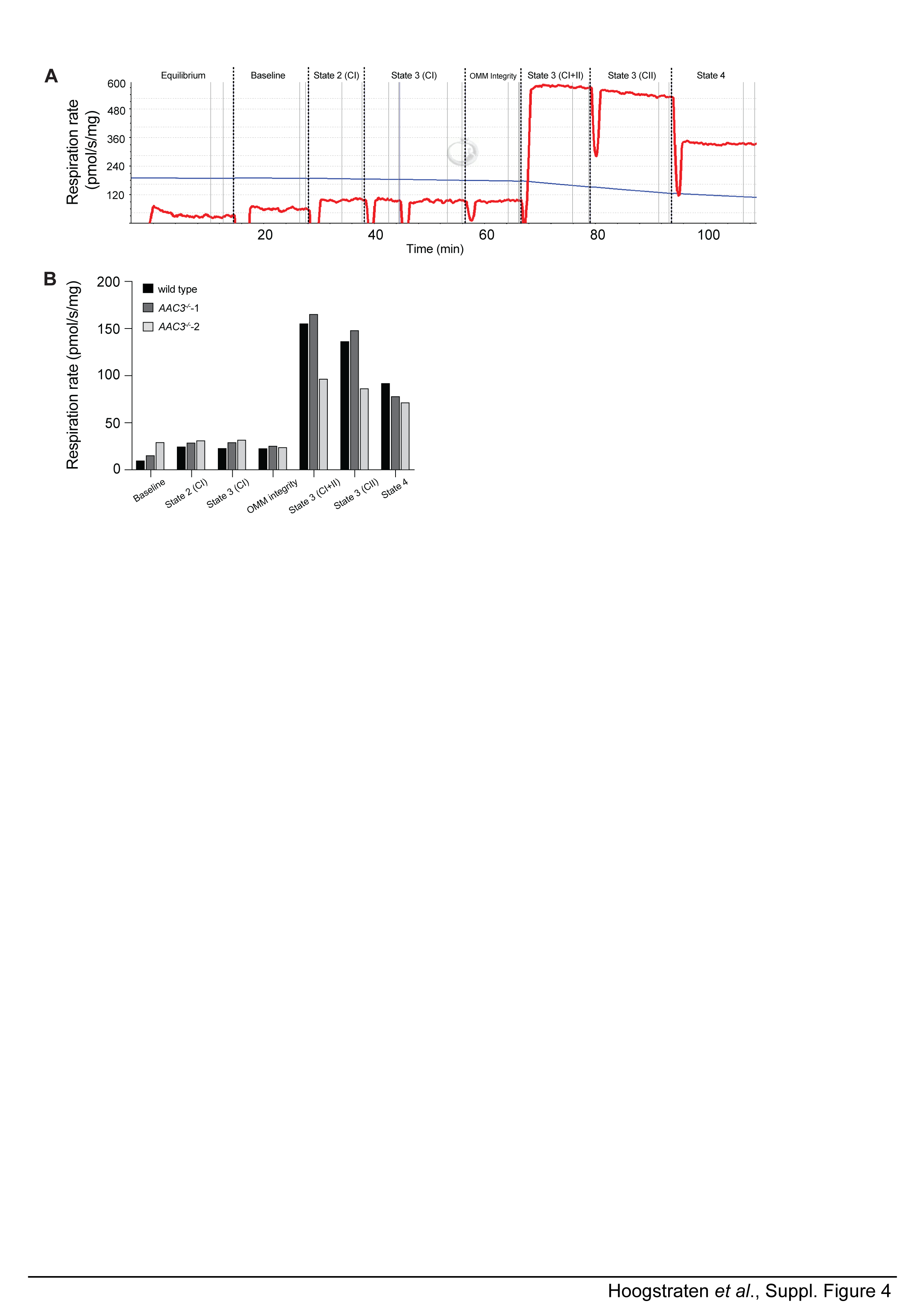

Supplement: Supplementary file 4 — Supplemental Figure 4 | Evaluating mitochondrial membrane integrity in isolated wild type and AAC3-/- mitochondria-enriched fractions by high-resolution respirometry. To ensure that mitochondrial membrane integrity was not lost due to sample preparation, wild type (black) and AAC3-/- (dark and light grey) ciPTEC were matured and mitochondria-enriched fractions were obtained by homogenizing cells and differential centrifugation, as described. Mitochondrial oxidative capacity and integrity of the mitochondrial membrane of these isolated fractions were evaluated using the high-resolution Oxygraph-2k respirometer, provided with Datlab 5 recording and analysis software (Oroboros Instruments, Innsbruck, Austria). A previously published protocol was followed to specifically determine mitochondrial membrane integrity after the isolation procedure (Lanza and Nair 2009). In two thermostated chambers (37°C), isolated mitochondria were added for each cell line, followed by evaluation of substrate-specific effects on cellular respiration. In short, baseline mitochondrial respiration represented the consumption of oxygen in absence of exogenous substrates. Addition of glutamate (10 mM, # G1251, Sigma-Aldrich) and malate (2mM, # M1000, Sigma-Aldrich) reflected oxygen consumption at OXPHOS complex I (State 2). Subsequent injections of saturating ADP levels (4 mM total, # A4386, Sigma-Aldrich) maximally stimulated complex I-driven respiration (State 3). Cytochrome C (10 μM, # C2506, Sigma-Aldrich) was then injected as a quality control to confirm the integrity of the outer mitochondrial membrane (OMM), followed by succinate (10 mM, # S2378, Sigma-Aldrich) to also stimulate complex II (State 3, CI+II) and rotenone (0.5 μM, # R8875, Sigma-Aldrich) to inhibit complex I and to measure complex II-driven respiration (State 3, CII). Lastly, oligomycin (2.5 μM, # O4876, Sigma-Aldrich) was added to inhibit ATP synthase and determine a proton leak across the inner mitochondrial membrane (Stat [file 204_2023_3510_MOESM4_ESM.tif]

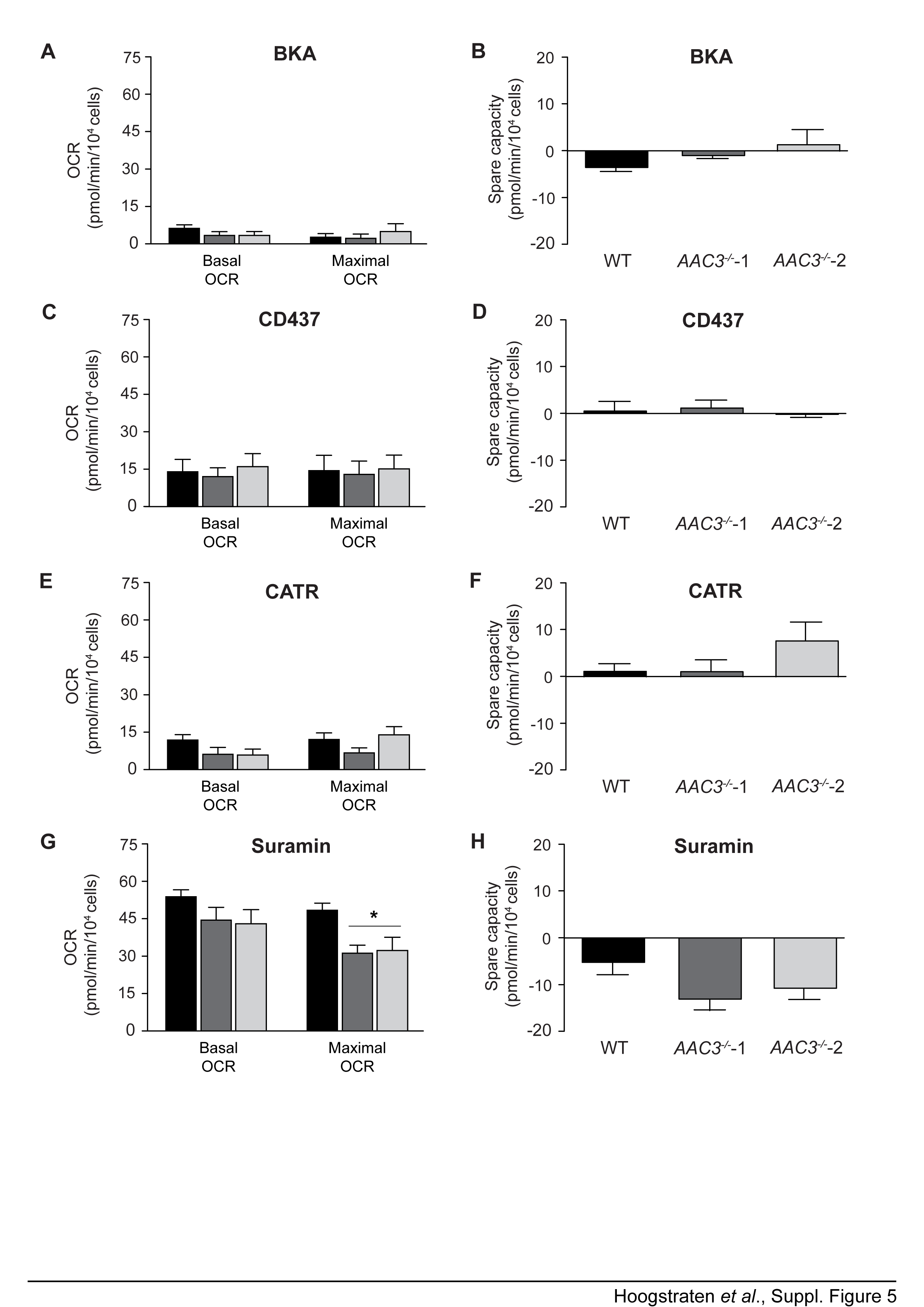

Supplement: Supplementary file 5 — Supplemental Figure 5 | Genetic AAC3 inhibition does not increase sensitivity of cells for AAC inhibitors. Mature wild-type ciPEC-OAT1 cells (black), and generated AAC3 knockouts (AAC3-/--1, dark grey and AAC3-/--2, light grey) were exposed to 100 µM AAC inhibitors BKA (A and B), CD437 (C and D), CATR (E and F), suramin (G and H) or 0.1% DMSO in medium containing 10 mM galactose for 12 hours at 37°C and 5% (v/v) CO2, followed by evaluation of mitochondrial respiration using the Seahorse XF Analyzer. Oxygen consumption rates (OCR) for all mitochondrial complexes were investigated. Data shown represents basal and maximal respiration (left) and spare capacity (right). Data was corrected for cell count, assessed by fluorescence microscopy after Hoechst staining. Significance was determined by one-way ANOVA with Dunnett’s post hoc analysis, *p<0.05, **p<0.01, ***p<0.001 and ****p<0.0001, mean ± SEM, N=3 independent experiments. Wild-type data is also presented in figure 5. (TIF 26793 KB) [file 204_2023_3510_MOESM5_ESM.tif]
